# The Redundancy of Peptidoglycan Carboxypeptidases Ensures Robust Cell Shape Maintenance in *Escherichia coli*

**DOI:** 10.1128/mBio.00819-16

**Published:** 2016-06-21

**Authors:** Katharina Peters, Suresh Kannan, Vincenzo A. Rao, Jacob Biboy, Daniela Vollmer, Stephen W. Erickson, Richard J. Lewis, Kevin D. Young, Waldemar Vollmer

**Affiliations:** a Centre for Bacterial Cell Biology, Institute for Cell and Molecular Biosciences, Newcastle University, Newcastle upon Tyne, United Kingdom; b Department of Microbiology and Immunology, University of Arkansas for Medical Sciences, Little Rock, Arkansas, USA; c Department of Biostatistics, University of Arkansas for Medical Sciences, Little Rock, Arkansas, USA

## Abstract

Peptidoglycan (PG) is an essential structural component of the bacterial cell wall and maintains the integrity and shape of the cell by forming a continuous layer around the cytoplasmic membrane. The thin PG layer of *Escherichia coli* resides in the periplasm, a unique compartment whose composition and pH can vary depending on the local environment of the cell. Hence, the growth of the PG layer must be sufficiently robust to allow cell growth and division under different conditions. We have analyzed the PG composition of 28 mutants lacking multiple PG enzymes (penicillin-binding proteins [PBPs]) after growth in acidic or near-neutral-pH media. Statistical analysis of the muropeptide profiles identified dd-carboxypeptidases (DD-CPases) that were more active in cells grown at acidic pH. In particular, the absence of the DD-CPase PBP6b caused a significant increase in the pentapeptide content of PG as well as morphological defects when the cells were grown at acidic pH. Other DD-CPases (PBP4, PBP4b, PBP5, PBP6a, PBP7, and AmpH) and the PG synthase PBP1B made a smaller or null contribution to the pentapeptide-trimming activity at acidic pH. We solved the crystal structure of PBP6b and also demonstrated that the enzyme is more stable and has a lower *K_m_* at acidic pH, explaining why PBP6b is more active at low pH. Hence, PBP6b is a specialized DD-CPase that contributes to cell shape maintenance at low pH, and *E. coli* appears to utilize redundant DD-CPases for normal growth under different conditions.

## IMPORTANCE

*Escherichia coli* requires peptidoglycan dd-carboxypeptidases to maintain cell shape by controlling the amount of pentapeptide substrates available to the peptidoglycan synthetic transpeptidases. Why *E. coli* has eight, seemingly redundant dd-carboxypeptidases has remained unknown. We now show that one of these dd-carboxypeptidases, PBP6b, is important for cell shape maintenance in acidic growth medium, consistent with the higher activity and stability of the enzyme at low pH. Hence, the presence of multiple dd-carboxypeptidases with different enzymatic properties may allow *E. coli* to maintain a normal cell shape under various growth conditions.

## INTRODUCTION

Bacteria are capable of growing in various environments or media that differ in the concentration of solutes, buffer compounds, or ions or in osmolarity or pH. For example, the Gram-negative bacterium *Escherichia coli* maintains robust growth under conditions of high and low osmolarity, acidic to alkaline pH, and minimal to nutrient-rich media (1). The cell buffers its cytoplasm by controlling the transport of molecules across its cytoplasmic membrane. However, the periplasm of *E. coli* is less buffered and is affected more by the environment than the cytoplasm; consequently, the pH of the periplasm is similar to that of the growth medium. Furthermore, many molecules with a mass of less than ~600 Da can diffuse into the periplasm via the outer membrane porins (2). Hence, the biogenesis of essential cell envelope components, such as peptidoglycan (PG) or lipopolysaccharide (LPS), must be sufficiently robust to enable the cell to grow under different pH and osmolyte conditions. How this robustness is achieved is largely unknown.

PG comprises glycan chains that are connected by short peptides, forming a net-like layer surrounding the cytoplasmic membrane (3). The PG layer protects the cell from bursting due to its turgor and maintains the structural integrity and the shape of the cell. In *E. coli*, a multitude of enzymes and regulatory proteins enlarge and remodel the PG layer when the cell is growing and dividing (4). PG synthesis is the principal target of important classes of antibiotics such as the β-lactams (e.g., penicillin) and glycopeptides (e.g., vancomycin).

The penicillin-binding proteins (PBPs), which are targeted by β-lactams, play a major role in the synthesis and remodeling of PG (5). The synthetic PBPs are either bifunctional glycosyltransferases/transpeptidases (class A PBPs; in *E. coli*, PBP1A, PBP1B, and PBP1C) or monofunctional transpeptidases (class B PBPs; in *E. coli*, PBP2 and PBP3) (5, 6). The activities of the synthetic PBPs are regulated by proteins associated with the intracellular cytoskeleton and, in *E. coli*, by outer membrane-anchored lipoproteins; PG synthesis is also coordinated with outer membrane constriction during cell division (7–12). According to a current model, PG synthases participate in dynamic multienzyme complexes, the elongasome and divisome, which facilitate PG growth at the side wall and cell division site, respectively (4, 13, 14).

Purified PBP1A and PBP1B polymerize the PG precursor lipid II *in vitro* (15, 16), but these PG synthases also exhibit hydrolytic dd-carboxypeptidase (DD-CPase) activity, trimming pentapeptides to tetrapeptides, particularly in the presence of their cognate Lpo activators and at low pH (17). It has been proposed that this DD-CPase activity rescues a synthetic class A PBP from being inactivated should it bind a donor peptide in the absence of an available acceptor peptide (17). However, the main peptide-trimming activity in *E. coli* stems from its eight class C PBPs, which are DD-CPases and/or dd-endopeptidases (DD-EPases): PBP4 (18), PBP4b (19), PBP5 (20–22), PBP6a (formerly known as PBP6) (20), PBP6b (23), PBP7 (24), AmpC (25), and AmpH (25, 26). The reason for this redundancy is unknown, but it may reflect the importance of DD-CPases in controlling the amount of pentapeptide donor substrates for the transpeptidation reaction of class A and B PBPs (27).

Many strains with multiple PBP deletions grow with few or no morphological defects, revealing a remarkable robustness of the PG synthetic machinery, at least under laboratory conditions (28). The DD-CPases are dispensable for the survival of *E. coli*, and, with the exception of PBP5, the deletion of the corresponding genes does not affect cell growth or morphology. The main DD-CPase of *E. coli* is PBP5, because the deletion of its gene, *dacA*, and of at least two other DD-CPase genes (or of *mrcA*, the PBP1A gene) causes aberrant cell morphologies with kinks, bends, or branches, and these defects can be corrected by ectopic expression of PBP5 but not by expression of one of the other DD-CPases (21, 29). The importance of PBP5 might be a consequence of the timing and the level of its expression: PBP5 is expressed primarily during early exponential growth, while PBP6a and PPB6b are expressed in the stationary and mid-exponential-growth phases, respectively (23, 30, 31). Importantly, ribosome profiling showed a significantly higher synthesis rate for PBP5 (~4,000 molecules synthesized per generation; MOPS [morpholinepropanesulfonic acid] complete medium, pH 7.4) than for PBP6, AmpC, and AmpH (all three between 400 and 700 molecules) or for PBP4b and PBP6b (both between 20 and 30 molecules) (32).

PBP5, PBP6a, and PBP6b are periplasmic proteins binding to the cytoplasmic membrane by a C-terminal amphiphilic α-helix, which is important for the physiological function of PBP5 (33). The membrane anchor is also important for PBP5 localization at the cell division site, which depends on ongoing PG synthesis (34). Adjacent to the membrane anchor, there is an elongated, β-sheet-rich domain, which appears to position the globular, catalytic domain at a fixed distance from the membrane (35–37). The region from amino acid 200 to amino acid 219 around the KTG motif of the CPase domain is important for the cell shape maintenance function of PBP5 (29). Although PBP5 and PBP6a have similar structures, PBP6a exhibits DD-CPase activity that is 5 times lower than that of PBP5 against the artificial substrate N^α^,N^ε^-diacetyl-Lys-d-Ala-d-Ala (AcLAA) and lacks activity against larger substrates (38), presumably explaining why PBP6a cannot compensate for the loss of PBP5 in the cell (22, 29, 33).

Here, we addressed the issue of why *E. coli* has so many redundant PG DD-CPases. We have analyzed the PG composition of 28 multiple PBP mutants grown at pH 7.5 or 5.0. PBP6b is the predominant CPase at acidic pH, consistent with our findings that the expression of PBP6b is enhanced at low pH and that PBP6b contributes to proper cell shape under this condition. PBP6b is more active and more stable at low pH than at neutral pH, and we present its crystal structure. Taken together, our data indicate that PBP6b is a DD-CPase specialized for growth at acidic pH and that the redundancy of DD-CPases ensures that sufficient activity is available under various pH conditions.

## RESULTS

### Muropeptide analysis reveals PBP activities in cells growing at different pH values.

To provide insight into the roles of *E. coli* PBPs that, as yet, have no known biological function, we determined the compositions of PG muropeptides from 28 *E. coli* strains that lacked various combinations of PBPs. Each strain was grown at both pH 5.0 and pH 7.5, and the results were analyzed to determine the changes associated with the presence and absence of different PBPs (see Table S1 in the supplemental material). In cells grown at pH 7.5, the presence of PBP5, PBP6b, or PBP7 was associated with higher DD-CPase activity; that is, the quantity of pentapeptide side chains in PG decreased while that of the tetrapeptides increased (see Fig. S1A and B and Table S1 in the supplemental material). PBP5 is the classic PG DD-CPase (27), and the muropeptide composition of strains containing PBP5 was consistent with this activity. Although PBP6a is most closely related to PBP5, PBP6a exhibited little or no DD-CPase activity *in vitro* (38). Consistent with these results, PBP6a was associated with only slight changes in muropeptide composition in cells grown at pH 7.5 or pH 5.0 (see Fig. S1 and Table S1). In fact, the presence of PBP6a was associated with muropeptide changes that were the opposite of those expected of a DD-CPase; in isogenic strains, the presence of PBP6a increased the percentage of pentapeptides (by up to 15%) and decreased the percentage of tetrapeptides (by as much as 12%) (see Fig. S1A and B and Table S1). These results were exactly the opposite of those associated with PBP5. Thus, PBP6a appears to be inactive as a DD-CPase and may even protect some pentapeptides from being cleaved.

In strains lacking PBP5, PBP4 appeared to be a strong DD-CPase at pH 7.5 (see Fig. S2A and B and Table S1). The DD-CPase activity associated with PBP7 was a surprise, because this enzyme is an endopeptidase (24) for which no DD-CPase activity had been described previously until a recent report describing its orthologue in *Pseudomonas aeruginosa* was published (39). Finally, the presence of PBP1B was also associated with weak DD-CPase activity at pH 5.0 (see Fig. S1C and D, S2C and D, and Table S1), which is consistent with the recently reported DD-CPase activity of the purified enzyme (17).

### PBP6b is the major DD-CPase at low pH.

**FIG 1:**
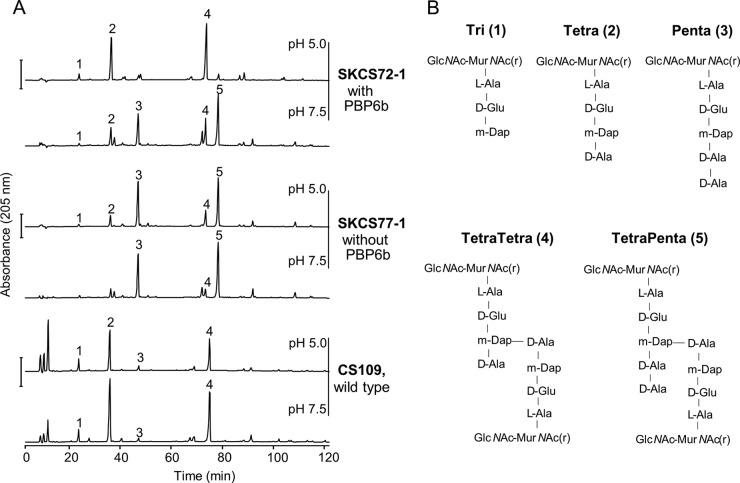
PBP6b is active in cells growing at pH 5.0. (A) Muropeptide profiles of *E. coli* wild-type strain CS109 and of two PBP mutants, SKCS72-1 (PBP6b^+^) and SKCS77-1 (PBP6b^−^), grown at pH 7.5 or 5.0. SKCS77-1 lacks PBP1A, PBP4, PBP4B, PBP5, PBP6a, PBP7, AmpH, AmpC, and PBP6b; SKCS72-1 lacks PBP1A, PBP4, PBP4B, PBP5, PBP6a, PBP7, AmpH, and AmpC. The main muropeptides are numbered. Bar, 500 mAU. (B) Names and structures of the muropeptides numbered in panel A. GlcNAc, N-acetylglucosamine; MurNAc(r), reduced N-acetylmuramic acid; m-Dap, mesodiaminopimelic acid.

PBP6b (also known as DacD) is another putative DD-CPase in *E. coli*, with 47% sequence identity to both PBP5 and PBP6a (23). The biological role of PBP6b is unknown, and previous experiments have identified only one phenotype associated with its absence, a delay in the biogenesis of the GOB-18 metallo-β-lactamase (40). When PBP6b was present in cells grown at pH 5.0, pentapeptides disappeared and tetrapeptides appeared, indicating that PBP6b exhibited strong DD-CPase activity at this pH (Fig. 1; see also Fig. S1C and D and Table S1 in the supplemental material). In fact, the activities of PBP4, PBP5, and PBP7 disappeared at pH 5.0, and PBP6b became by far the most prominent DD-CPase, with inferred activity equivalent to that of PBP5 at pH 7.5 (see Fig. S2C and D and Table S1).

In order to better characterize the activity of PBP6b *in vivo*, we next compared the muropeptide composition of *E. coli* SKCS77-1, a strain that lacks all class C PBPs and the bifunctional class A PBP1A, to that of an isogenic mutant, SKCS72-1, which contains PBP6b (Fig. 1; see also Table S1 in the supplemental material). Pentapeptides were highly abundant in SKCS77-1 muropeptides at both pH values (Fig. 1), consistent with the absence of all DD-CPases in this strain. In contrast, some DD-CPase activity of PBP6b was observed at pH 7.5 in SKCS72-1 (Fig. 1; see also Table S1). At this pH, in the presence of PBP6b, the fraction of pentapeptides decreased by a quarter (from ~56% to ~42%), while the fraction of tetrapeptides increased by almost one-third (from ~38% to ~50%). Interestingly, the tetrapeptide-rich muropeptide profile of SKCS72-1 was restored by growth at pH 5.0 (Fig. 1) to the lower levels typical for the wild-type strain. The fraction of pentapeptides decreased by 90% (from ~53% to ~5%), and the fraction of tetrapeptides doubled (from ~40% to ~82%) (see Table S1), consistent with a significant increase in PBP6b DD-CPase activity at pH 5.0.

Overall, these data show that PBP6b was an active DD-CPase in cells grown at pH 5.0, under which condition it trimmed the majority of pentapeptides, though it was also present and partially active at pH 7.5 when all other LMW PBPs were absent.

### *dacD* transcription and translation increase at pH 5.0.

**FIG 2:**
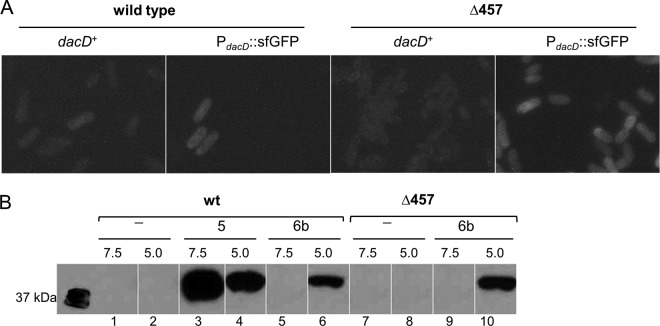
Increased transcription and translation of *dacD* at pH 5. (A) *dacD* transcription. Chromosomal *dacD* was replaced with the gene encoding superfolder GFP (sfGFP) and placed under control of the *dacD* promoter (P_*dacD*_::sfGFP). The wild-type parent (CS109) and a mutant lacking PBP4, PBP5, and PBP7 (Δ457 strain; CS315-1) with and without *dacD*::sfGFP were grown to an OD_600_ of 0.7 to 0.8 in Penassay broth at pH 5.0 and observed for fluorescence. (B) Production of PBP6b and PBP5. Genes encoding C-terminal FLAG fusions to PBP5 (PBP5-FLAG) or PBP6b (PBP6b-FLAG) were inserted into their natural chromosomal positions. Cells with and without the fusions were grown in Penassay broth at pH 7.5 and pH 5.0, and the FLAG-tagged proteins from membrane fractions (10 µg) were separated by SDS-PAGE and detected by Western blotting, using HRP-conjugated anti-FLAG antibody. wt, wild-type *E. coli* CS109; Δ457, *E. coli* CS315-1 (a mutant lacking PBP4, PBP5, and PBP7). Lanes 1 and 2, wild-type cells; lanes 3 and 4, wild-type cells expressing PBP5-FLAG; lanes 5 and 6, wild-type cells expressing PBP6b-FLAG; lanes 7 and 8, Δ457 mutant cells only; lanes 9 and 10, Δ457 mutant cells expressing PBP6b-FLAG. Cells were grown at either pH 7.5 or pH 5.0, as indicated above the lanes.

The enhanced *in vivo* DD-CPase activity of PBP6b at pH 5.0 could be explained by an increase in PBP6b protein levels and/or by an increase in the specific activity of PBP6b at this pH. Indeed, ribosome profiling indicates that *E. coli* MG1655 produces only 29 molecules of PBP6b per generation when grown in a rich medium or only 6 molecules per generation when grown in a minimal MOPS medium at pH 7.4 (32). Hence, it was feasible that PBP6b was not produced at pH 7.5 but was made in a substantial quantity only at pH 5.0. We therefore measured the influence of pH on *dacD* expression by replacing the chromosomal *dacD* open reading frame with the superfolder GFP gene (*sfgfp*), thereby placing it under control of the native *dacD* promoter and ribosome binding site. The fusion was subcloned into the CS109 parental strain and into the CS315-1 PBP mutant (ΔPBP4, PBP5, and PBP7). When the sfGFP signal per cell was quantified by flow cytometry (Table 1), there was little difference in the amounts of sfGFP produced when either strain was grown at pH 7.5, even when the cells contained the *dacD-sfgfp* reporter fusion (Table 1; pH 7.5 values). Therefore, very little sfGFP (and, by extension, PBP6b) was produced at pH 7.5. At pH 5.0, control strains without the sfGFP fusion exhibited slightly higher background fluorescence (Fig. 2A, *dacD*-positive [*dacD*^+^] frames; Table 1, pH 5.0, lines 1 and 3). In contrast, when the latter two strains carried the *dacD-sfgfp* reporter fusion, the amount of sfGFP increased noticeably (Fig. 2A). When background fluorescence is subtracted, the calculated amount of GFP increases approximately 25- to 40-fold in both SKCS143-1 (the wild type) and SKCS144-1 (the triple PBP mutant) (Table 1, pH 5.0, lines 2 and 4). Thus, growth at pH 5.0 strongly induced gene expression from the *dacD* promoter.

**TABLE 1:** *DacD*, transcription increases at pH 5.0^a^

Strain	PBPs deleted	P_*dacD*_:: sfGFP	Mean GFP intensity/cell (AU)	
			pH 7.5	pH 5.0
CS109 (wt)	None	−	68	129
SKCS143-1	None	+	72	247
CS315-1	4, 5, 7	−	93	137
SKCS144-1	4, 5, 7	+	107	257

a Strains with and without P_*dacD*_::sfGFP were grown in Penassay broth at pH 7.5 and pH 5.0. For each strain, the GFP signal intensity of 100,000 cells was measured by fluorescence-activated cell sorter (FACS) analysis and is reported in arbitrary units (AU). An increase in GFP intensity denotes increased *dacD* transcription. The experiment was repeated at least twice, with equivalent results.

To determine whether the increase in *dacD* transcription also led to an increase in PBP6b protein levels at pH 5.0, FLAG gene sequences were fused to the 3′ termini of the chromosomal genes encoding PBP6b (*dacD*) and PBP5 (*dacA*). Both PBP6b-FLAG and PBP5-FLAG behaved like wild-type proteins in that their expression in the CS109 parent caused no growth or morphological phenotypes, while their expression in the CS315-1 PBP mutant complemented the morphological defects of this strain (data not shown). Neither untagged PBP5 nor PBP6b bound the anti-FLAG antibody at either pH (Fig. 2B, lanes 1, 2, 7, and 8). About 25% less PBP5-FLAG was produced in cells grown at pH 5.0 than in cells grown at pH 7.5 (Fig. 2B, lanes 3 and 4), suggesting either that *dacA* expression was inhibited or that PBP5 was less stable at pH 5.0. In contrast, the PBP6b-FLAG band was present only when the parent or the mutant was grown at pH 5.0 (Fig. 2B, lanes 6 and 10) and was undetectable in cells of either strain grown at pH 7.5 (Fig. 2B, lanes 5 and 9), consistent with the low copy number determined by ribosome profiling (32). The amount of PBP6b-FLAG at pH 5.0 was ~22% of the amount of PBP5 in cells grown at pH 7.5 (Fig. 2B and data not shown). Since PBP5 is present in 800 to 4,000 copies per cell (32, 41), we estimate that 200 to 800 copies of PBP6b were produced at pH 5.0. Given that there are 6 to 30 copies of PBP6b per cell at pH 7.5 (32), the amount of PBP6b expanded by 30- to 100-fold in cells grown at pH 5.0. Transcription increased by about 25- to 40-fold (as described above), so both methods corroborate a similar and significant increase in the levels seen with PBP6b at pH 5.0. In addition, there was ~50% more PBP6b in the triple PBP mutant CS315-1 (Fig. 2B, lane 10), suggesting that PBP6b might be upregulated in the absence of PBP4, PBP5, and PBP7. In summary, the production of PBP6b was below detectable levels when cells were grown at pH 7.5, but the protein was expressed in much greater amounts in cells grown at pH 5.0, consistent with the idea that low pH induces *dacD* gene expression and PBP6b production.

### PBP6b has higher activity at acidic pH.

**FIG 3:**
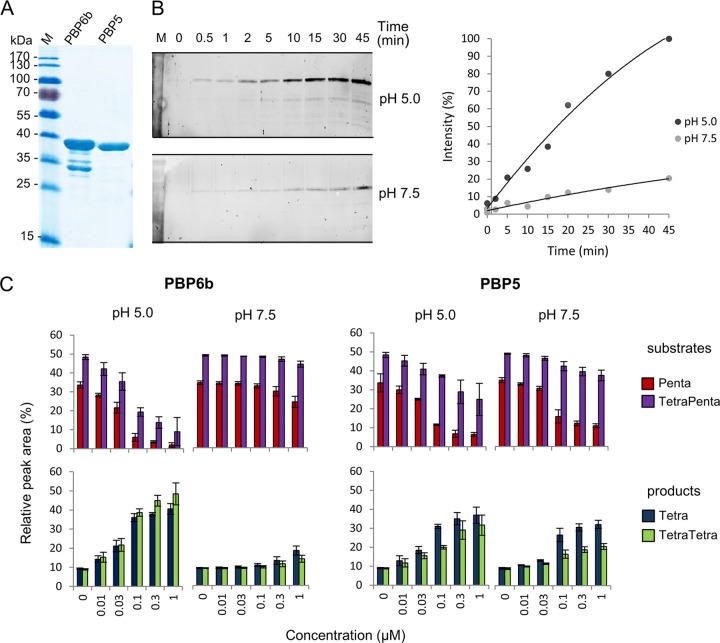
Activities of PBP6b and PBP5. (A) Purified PBP6b and PBP5 (2.7 µg) were analyzed by SDS-PAGE and Coomassie staining. (B) Increased Bocillin binding of PBP6b at pH 5.0. Purified PBP6b was incubated with the fluorescent penicillin derivative Bocillin for different time periods at 37°C and pH 5.0 or pH 7.5. The intensities of the fluorescence signals were quantified with ImageJ and are given relative to the value at pH 5.0 (45 min). (C) Activity of PBP6b and PBP5 against pentapeptide-rich PG from *E. coli* CS703-1 at pH 7.5 or 5.0. PG was incubated with different concentrations of PBP6b and PBP5, followed by digestion with cellosyl, reduction, and muropeptide analysis. Only changes in the main substrate (Penta, TetraPenta) and product (Tetra, TetraTetra) muropeptides are summarized. Representative chromatograms are shown in Fig. S3 and S4 in the supplemental material. The values are means ± standard deviations (SD) of the results of 3 independent experiments.

We purified PBP6b and PBP5 (Fig. 3A) lacking their signal peptides but containing an N-terminal oligohistidine tag, which was subsequently removed by proteolytic cleavage. Both proteins contained the α-helical membrane anchor near the C terminus. We used several assays to test the activity of purified PBP6b under different pH conditions. First, we measured the binding of the fluorescent penicillin derivative Bocillin-FL. PBP6b was incubated with Bocillin-FL at 37°C and pH 5.0 or pH 7.5 for different time periods, followed by gel electrophoresis and fluorescence detection of the PBP6b-Bocillin FL complex. PBP6b bound Bocillin-FL significantly faster at pH 5.0 than at pH 7.5 (Fig. 3B).

Second, we assayed the DD-CPase activities of PBP6b and PBP5 against pentapeptide-rich PG from *E. coli* CS703-1 at pH 5.0 and 7.5. The PG was incubated with increasing concentrations of PBP6b or PBP5 for 1 h at 37°C, followed by digestion with the muramidase cellosyl and analysis of the muropeptide profile by high-pressure liquid chromatography (HPLC); representative chromatograms are shown in Fig. S3 to S4 in the supplemental material. PBP6b produced significant amounts of tetrapeptides in a concentration-dependent manner at pH 5.0 but not at pH 7.5 (Fig. 3C; see also Fig. S3), consistent with the enhanced activity of PBP6b in cells growing at acidic pH (Fig. 1). In contrast, PBP5 was active at both pH values, with slightly higher activity at pH 5.0 than at pH 7.5 (Fig. 3C; see also Fig. S4). The activity of PBP5 was greater against monomeric pentapeptides than against dimeric (cross-linked) tetrapentapeptides, whereas PBP6b had similar activities against monomers and dimers. To determine if PBP6b and PBP5 act on muropeptides with a Gly residue at position 5 (instead of d-Ala), which are rare in *E. coli* but abundant in other species such as *Caulobacter crescentus*, each enzyme was incubated with pentapeptide- and glycine-rich PG from *C. crescentus* at different pH values, followed by muropeptide analysis; representative chromatograms are shown in Fig. S5A. PBP6b showed significant activity only against the *C. crescentus* PG at pH 5.0, whereas PBP5 was active at both pH values (see Fig. S5B), which was similar to what we observed for *E. coli* PG (Fig. 3C). PBP6b and PBP5 were both less active against glycine-containing pentapeptides (Penta-Gly_5_ and TetraPenta-Gly_5_) than against normal pentapeptides (Penta and TetraPenta), which is consistent with the observation that the low levels of Gly-containing pentapeptides were similar in strains with or without PBP6b or PBP5 grown at both pH values (see Table S1).

### Crystal structure of *E. coli* PBP6bΔC.

To rationalize the molecular basis of the pH dependency of the DD-CPase activity of PBP6b, its crystal structure was solved by molecular replacement using the apo structure of PBP5 (PDB identifier [PDBid] 1NZO) as the search model (37) and refined to a resolution of 2.4 Å. Data collection details and model refinement statistics are summarized in Table S3 in the supplemental material. The construct that yielded crystals suitable for structure determination (PBP6bΔC) lacked the N-terminal 21 residues of the signal peptide and the C-terminal 14-residue membrane anchor. The structure of PBP6bΔC comprises two domains; the CPase domain is formed of residues Glu2 to Thr262, whereas the C-terminal domain comprises Thr263 to Thr346, with two breaks in the modeled polypeptide chain, between residues Lys272 and Asp287 and between residues Leu317 and Pro320, because of disorder. The crystals contain two PBP6bΔC copies per asymmetric unit, which can be superimposed with the root mean square deviation (RMSD) of 0.3 Å on 324 aligned Cα atoms. There is no significant difference in the relative dispositions of the two domains in the two molecules in the asymmetric unit, therefore, the two chains can be considered to be essentially identical.

Given the 48% sequence identity between the PBP6b and PBP5 (see Fig. S6 in the supplemental material), it is unsurprising that the structures of PBP6bΔC and various PBP5ΔC constructs superimpose well. A global superimposition of 292 Cαs yields an RMSD value of 1.4 Å, indicating that the relative dispositions of the two domains in each PBP are the same. The CPase domains superimpose on 256 aligned Cα atoms with an RMSD of 1.1 Å, whereas the C-terminal domains superimpose with an RMSD of 2.2 Å on 70 aligned Cαs. The C-terminal domains share sequence identity of 34%, consistent with their poorer structural alignment. Furthermore, the C-terminal domain in PBP6bΔC is relatively poorly ordered, with an average B factor on main chain atoms of 59.3 Å^2^ compared to 31.5 Å^2^ for the CPase domain, whereas the B factor distributions for PBP5 (PDBid 1NZO) are more even, with equivalent values of 29.9 Å^2^ and 32.1 Å^2^, respectively. Despite cocrystallization of PBP6bΔC with 2 mM diacetyl-l-Lys-d-Ala-d-Ala, there is no evidence of bound product from the pseudosubstrate. The crystal packing involves the region immediately surrounding the PBP6bΔC active site (Ser42) such that the observed crystal packing would likely have been disfavored had the product of the CPase reaction been bound.

### Molecular explanation of the pH dependency on activity for *E. coli* PBP6b.

Does the structure of PBP6bΔC explain the pH dependency on enzyme activity? The nature of the residues in the immediate vicinity of Ser42 compared to PBP5, which does not display the same pH effects, may provide the answer to this question. Of all the residues within 10 Å of the Ser42 side chain hydroxyl, all but one are invariant between PBP6bΔC (Asn39) and PBP5 (Asp49). While asparagine does not normally contribute directly to catalysis, unlike aspartate, which can act as a general acid or a base, the amide-imide tautomerization of Asn92 in the glycoside hydrolase family 45 endocellulase has recently been described (42). In the endocellulase, Asn92 is on one side of the active site groove, 6 Å away from the general acid Asp114 on the other side of the groove that accommodates the cellulose substrate. As with PBP6b, there is strong dependency on acidic pH for activity of the wild-type cellulase, which is eliminated by mutating Asn92 to aspartate. Asn39 in PBP6bΔC is 9 Å away from the nucleophilic Ser42, and, should the CPase reaction proceed using a Grotthuss-like proton relay mechanism similar to that of the GH45 cellulase (42), residues His214 and Ser216 are likely intermediates between Ser42 and Asn39. Perhaps this mechanism is disfavored at neutral pH or in PBP5, where His216 and Asp218 are the intermediaries between Ser44 and Asp41.

**FIG 4:**
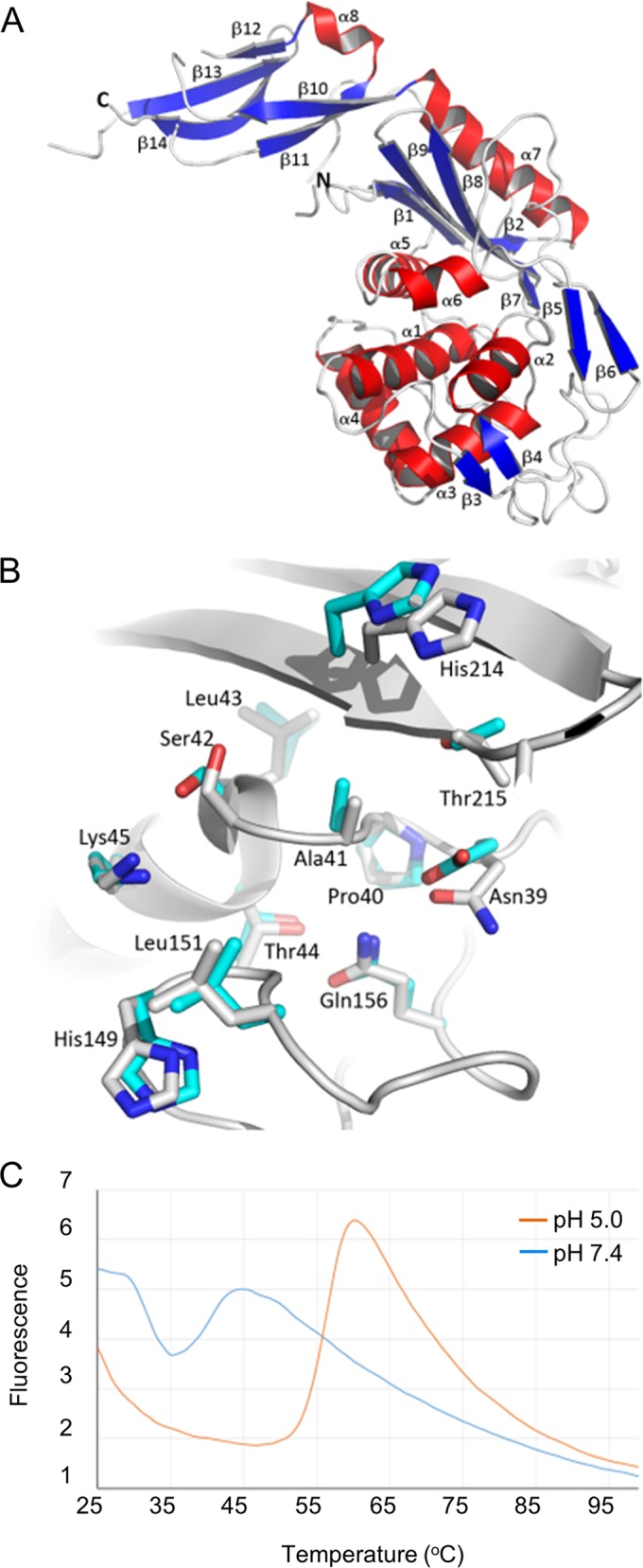
Crystal structure of PBP6bΔC. (A) The crystal structure of PBP6bΔC is depicted as a cartoon, with secondary structure elements colored blue (β-strands), red (α-helices), and white (loops). Each strand and helix is labeled. (B) A superimposition of the active sites of PBP6bΔC (white) and *E. coli* PBP5 (*Ec*PBP5) (PDBid 1NZO; cyan); labels correspond to the PBP6b sequence. Residues within 6 Å of the nucleophilic Ser42 are shown as sticks. (C) Differential scanning fluorimetry of PBP6b at pH 5.0 and 7.4 revealed a 16°C difference in *T_m_* at the 2 pH values.

Alternatively, the pH dependency of PBP6b might be explained by a change either in protein stability and/or in the *K_m_* for its substrate as a function of pH. Consequently, the melting temperature (*T_m_*) for PBP6b and PBP6bΔC was obtained by differential scanning fluorimetry (DSF) and by circular dichroism-based thermal melt analysis. For both proteins, both methods revealed a 16°C difference in *T_m_*, 57.5°C at pH 5.0 versus 41.5°C at pH 7.4 (Fig. 4C and data not shown). For PBP5, DSF was unsuccessful because the presence of the Triton X-100 detergent used in protein purification led to unacceptable background fluorescence. Circular dichroism-based thermal melts yielded *T_m_* values of 58.4°C and 51.2°C at pH 5.0 and 7.4, though precipitation of the protein during the course of the experiment hindered the collection of ideal data. Nevertheless, the similarity of the *T_m_* value at different pH values suggests that PBP5 is less prone to instability at pH 7.4 than PBP6b.

To complement the protein stability measurements, the PBP6b DD CPase activity was tested against diacetyl-l-Lys-d-Ala-d-Ala, and the kinetic parameters were measured under different pH conditions in a spectrophotometric d-alanine release assay. The *V*_max_ and *k*_cat_ values were similar at the two pH values (Table 2) and were consistent with published values measured at pH 8.5 (43). However, the *K_m_* value was 18-fold lower at pH 5.0 than at pH 7.5, which is consistent with the increased binding of Bocillin FL at this pH, indicating that the increased activity of PBP6b against pentapeptide-rich PG at acidic pH (Fig. 3) was due to both enhanced protein stability and enhanced substrate binding affinity. Without elucidation of structures of PBP6b at both pH values, however, a molecular explanation of the pH dependency on protein stability and *K_m_* cannot be provided.

**TABLE 2:** Kinetic parameters of PBP6b with the substrate diacetyl-l-Lys-d-Ala-d-Ala

pH	*K_m_* (mM)	*V*_max_ (µmol ⋅ min^−1^ ⋅ mg^−1^)	*k_cat_* (s^−1^)
5.0	1.57 ± 0.34	2.27 ± 0.25	1.57 ± 0.17
7.5	28.6 ± 1.90	1.35 ± 0.15	0.93 ± 0.10

### PBP6b, but not PBP5 or PBP6a, preserves wild-type morphology at low pH.

In the absence of PBP5, *E. coli* cells are abnormally shaped and their peptidoglycan contains more pentapeptides (21, 22, 44). However, those observations were made in cells grown in a rich medium at pH 7.0 to 7.5. As we show above, PBP6b cleaves pentapeptides more efficiently at pH 5.0, making it possible that PBP6b might replace PBP5 to produce cells having wild-type morphology under those conditions. To test this idea, cells lacking different PBPs were grown at pH 7.5 and 5.0, and alterations in cell shape were quantified by means of flow cytometry.

**FIG 5:**
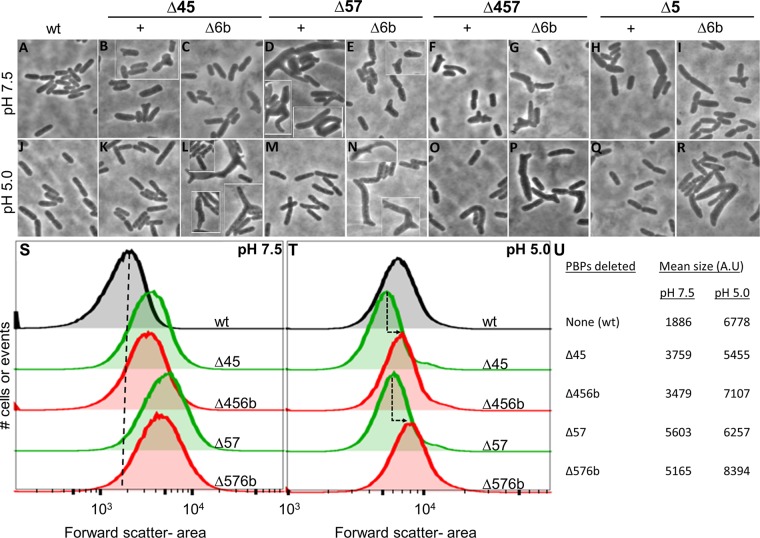
PBP6b (DacD) promotes normal morphology at pH 5.0. (A to R) Phase microscopy of *E. coli* DD-CPase mutants. Single, double, or triple PBP mutants with or without PBP6b were grown to an OD_600_ of 0.7 to 0.8 in Penassay broth at pH 7.5 (A to I) or pH 5.0 (J to R) and then imaged. Cells from separate visual fields are set off by white frames. Strains: CS109 (WT), CS219-1 (ΔPBP4 and PBP5), SKCS132-1 (ΔPBP4, PBP5, and PBP6b), CS204-1 (ΔPBP5 and PBP7), SKCS133-1 (ΔPBP5.7 and PBP6b), CS315-1 (ΔPBP4, PBP5, and PBP7), and SKCS127-1 (ΔPBP4, PBP5, PBP7, and PBP6b). (S and T) Histograms of cell populations sized by flow cytometry. PBP mutants (10^5^ cells from the experiments represented in panels A to R as described above) were grown at pH 7.5 (S) or pH 5.0 (T) and then analyzed by flow cytometry. The mean forward scatter area was plotted for each cell in the population. Each peak corresponds to the overall distribution of a single strain. Individual peaks were overlaid by using FlowJo software. Green peaks represent the immediate parent strain for each of the ΔPBP6b mutants, which are graphed in red. (S) The vertical black dashed line provides a visual reference point for the mean cell size of the wild-type population. (T) The dashed arrows indicate the deviation in cell size between a parent and its cognate PBP6b mutant. (U) Quantification of the shape defects for PBP6b mutants. The shape defects of mutants (shown in panels A to R) were analyzed by flow cytometry as described for panels S and T. The mean cell sizes at pH 7.5 and pH 5.0 are represented by the mean of the forward scatter area and are reported in arbitrary units (A.U).

As described before (21, 22), *E. coli* mutants lacking PBP5 exhibited aberrant morphologies when grown at pH 7.5, particularly in the absence of other low-molecular-weight (LMW) PBPs (compare Fig. 5A to the mutant cell shapes in Fig. 5B to I). These shape changes produced large rightward shifts in the forward light scatter of cell populations (Fig. 5S) and increased the average mean size of these cells (expressed in arbitrary units [AU]) (Fig. 5U). The magnitude of these alterations paralleled the subjective degree of morphological change as determined by microscopy. Notably, at pH 7.5, the presence or absence of PBP6b influenced cell shape very little (~10% variance) (Fig. 5B to I and U), reinforcing the conclusion that PBP5 is the major low-molecular-weight PBP that maintains cell shape.

In sharp contrast, when grown at pH 5.0, mutants lacking PBP5 but containing wild-type PBP6b produced cells with almost normal rod shapes (Fig. 5K and M and Q). These cells had population distributions (Fig. 5T, green peaks) and mean sizes (Fig. 5U) that were near or slightly smaller than those of the wild-type parent. However, strains lacking both PBP5 and PBP6b continued to produce morphologically aberrant cells (Fig. 5L and N and R). Compared to the results seen with matched strains containing wild-type PBP6b (Fig. 5T, green lines), the population distributions of cells lacking PBP5 and PBP6b shifted significantly rightward (Fig. 5T, red lines), and the mean cell sizes increased in the mutants (Fig. 5U). In short, in the absence of PBP5, PBP6b was required to maintain the normal rod shape of *E. coli* grown at pH 5.0.

**FIG 6:**
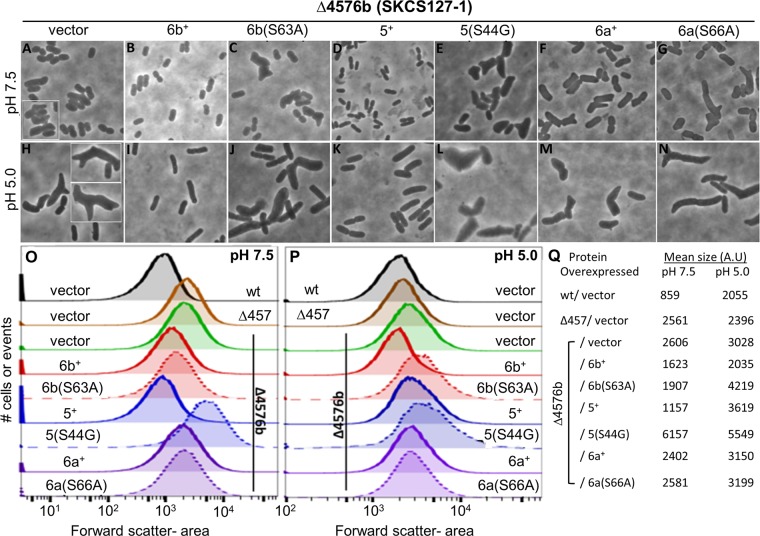
Functional PBP6b is required to maintain cell shape at pH 5.0. (A to N) Phase images of complementation with DD-CPases. An *E. coli* mutant lacking PBP4, PBP5, PBP7, and PBP6b (SKCS127-1) carried plasmid pBAD vector (A and H), PBP6b^+^ (B and I), PBP5^+^ (D and K), or PBP6a^+^ (F and M) or a plasmid encoding the corresponding inactive mutant proteins: PBP6b(S63A) (C and J), PBP5(S44G) (E and L), or PBP6a^+^(S66A) (G and N). Each strain was grown overnight in Penassay broth at pH 7.5 (A to G) or pH 5.0 (H to N) and diluted into fresh medium. Wild-type proteins or their inactive mutant versions were induced by adding 0.2% arabinose. Cultures were incubated to an OD_600_ of 0.7 to 0.8, harvested for flow cytometry, and imaged. Cells from separate visual fields are set off by white frames. (O and P) Histograms of cell populations sized by flow cytometry. Strains represented in panels A to N (10^5^ cells each) were grown at pH 7.5 (O) or pH 5.0 (P) and then analyzed by flow cytometry. The mean forward scatter area was plotted for each cell in the population. Each graph represents the overall distribution of a single strain as indicated. Individual peaks were overlaid by using FlowJo software. Continuous lines represent complementation with the active version of the respective PBPs. Dashed lines represent complementation with the inactive version of the respective PBPs. Colors represent the individual proteins as follows: red, PBP6b; blue, PBP5; purple, PBP6a. (Q) Quantification of shape defects for complemented strains. Complementation by wild-type and mutant PBPs was analyzed by flow cytometry. The mean cell sizes at pH 7.5 and pH 5.0 are represented by the forward scatter area and are reported in arbitrary units (A.U).

We next tested the function of PBP6b and closely related DD-CPases by producing these proteins from plasmid-borne clones. The host was *E. coli* strain SKCS127-1, a mutant with visibly aberrant morphology, whose cells exhibited short branches, kinks, and buds (see, e.g., Fig. 6A and H). At both pH 7.5 and pH 5.0, the population distribution of SKCS127-1 (Fig. 6O and P, solid green lines) skewed to the right of CS109 (Fig. 6O and P, wt), and the mean cell size increased (Fig. 6Q). Compared to the uncomplemented strain (Fig. 6H), supplying PBP6b to SKCS127-1 restored wild-type shape to cells grown at pH 5.0 (Fig. 6I), and the population distribution moved leftward (Fig. 6P, solid red line), to become equivalent to that of nonmutated *E. coli* (Fig. 6P, solid black line). In addition, the 3028 AU mean cell size of the parent (Fig. 6Q, pH 5.0, vector) decreased to 2,035 AU in the complemented strain (Fig. 6Q, pH 5.0, 6b^+^), equivalent to the 2,055 AU of nonmutated cells (Fig. 6Q, pH 5.0, wt). Thus, PBP6b effectively replaced the function of PBP5 in cells grown at pH 5.0, the condition under which PBP6b is most active.

Restoring PBP6b also returned the mutant to a more normal rod shape at pH 7.5 (compare Fig. 6B to Fig. 6A). The population distribution of the complemented strain (Fig. 6O, solid red line) was closer to that of wild-type cells (Fig. 6O, black line) than that of cells lacking PBP6b (Fig. 6O, solid green line), though shape recovery was not complete. Also, while the 2,606-AU mean cell size of SKCS127-1 (Fig. 6Q, pH 7.5) decreased to 1,623 AU in the complemented strain (Fig. 6Q, pH 7.5), this change did not reproduce the original size of 859 AU for wild-type *E. coli* (Fig. 6Q, pH 7.5, wt). We estimated the amount of PBP6b required to effect this change by using an enzyme-linked immunosorbent assay (ELISA) to detect the amount of fluorescently labeled PBP6b (sfGFP-PBP6b) (44). Roughly 12,000 to 15,000 molecules of sfGFP-PBP6b per cell were required to produce a more normal rod-shaped morphology in the absence of PBP5 (data not shown). For comparison, there are 800 to 4,000 molecules of PBP5 per cell under similar conditions (41, 45). Thus, although PBP6b was less active at pH 7.5, producing PBP6b in sufficient quantity could partially compensate for the loss of PBP5 at this pH.

To put the results reported above in better perspective, we expressed PBP5 and PBP6a in mutants lacking PBP5, to compare the effects of these closely related enzymes with those produced by PBP6b. As expected (21), at pH 7.5, supplying PBP5 in *trans* returned strain SKCS127-1 to almost normal morphology (Fig. 6D; Fig. 6O, solid blue line; and Fig. 6Q, 5^+^), but supplying wild-type PBP6a had no effect (Fig. 6F; Fig. 6O, solid purple line; and Fig. 6Q, 6^+^). These results are consistent with reports that PBP5 is an active DD-CPase whereas PBP6a has little or no such activity (38, 44). Supplying PBP5 to SKCS127-1 at pH 5.0 did produce a more uniform population of rod shaped cells (Fig. 6K), but these cells were wider than normal, and the mean cell size increased to 3,619 AU (Fig. 6Q, pH 5.0, 5^+^). Thus, at pH 5.0, PBP5 did not restore wild-type morphology to SKCS127-1. Ectopic PBP6a neither suppressed nor enhanced the shape defects of this mutant (Fig. 6M; Fig. 6P, solid purple line; and Fig. 6Q), consistent with this PBP having little or no DD-CPase activity. Thus, neither PBP5 nor PBP6a could substitute for the absence of PBP6b in cells grown at pH 5.0.

Finally, we determined if PBP6b needed to be enzymatically active to correct the morphological abnormalities of cells lacking PBP5. In a PBP5^−^ mutant grown at neutral pH, ectopic production of PBP6b reduces PG pentapeptide levels, whereas the enzymatically inactive missense variant PBP6b(S63A) has no effect (44). Therefore, we expressed the latter protein in SKCS127-1 to see if shape complementation required the activity of PBP6b. As noted above, cells lacking PBP6b were misshapen at pH 5.0 (see, e.g., Fig. 6H and P, solid green line), but expressing inactive PBP6b(S63A) in *trans* made these cells even more aberrant (Fig. 6J and P, dotted red line), with the mean cell size increasing from 3,028 to 4,219 AU (Fig. 6Q). The increase in shape diversity was even greater in cells expressing the enzymatically inactive PBP5(S44G) variant (Fig. 6L and P, dotted blue line), and the mean size of these cells reached 5,549 AU (Fig. 6Q, pH 5.0). This PBP5(S44G) effect was even more pronounced at pH 7.5 (Fig. 6E; Fig. 6O, dotted blue line; and Fig. 6Q). In contrast, the inactive variant PBP6a(S66A) had no effect on morphological diversity at either pH 7.5 or pH 5.0 (Fig. 6N; Fig. 6O and P, dotted purple line; and Fig. 6Q). Thus, expressing inactive forms of PBP6b or PBP5 failed to restore wild-type shape to cells lacking PBP5 and instead exacerbated their morphological defects. The results confirmed that enzyme activity was a primary cause of cell shape deficiencies.

Altogether, the results described above establish that at pH 5.0, active PBP6b is required to produce *E. coli* cells of wild-type shape. Furthermore, under these conditions, neither PBP5 nor PBP6a could fully substitute for PBP6b.

## DISCUSSION

The single-layered PG sacculus of *E. coli* needs to be enlarged and remodeled to allow cell growth; these processes take place in the periplasm and are therefore affected by environmental conditions such as the concentration of osmolytes and ions or the external pH. Hence, the PG synthesis and remodeling system must be sufficiently robust to function under different conditions. PG synthesis and hydrolysis are achieved by a multitude of enzymes (4). For example, *E. coli* has as many as 26 periplasmic PG hydrolases, including 8 DD-CPases, 6 DD-endopeptidases, 4 MurNAc-l-Ala amidases, and 8 lytic transglycosylases (46). The reason for this redundancy of PG hydrolases is not well understood.

### Dissecting enzyme function in redundant pathways.

In processes with high redundancy, such as PG hydrolysis, it can be challenging to dissect the individual roles for each component. Chemical genomics screens can measure the fitness phenotypes of single mutants under hundreds of different growth conditions, revealing the most important genes, but system redundancy may prevent function determination for minor genes (32). To better understand the role of redundant genes, the corresponding proteins can be purified and their activity measured under conditions similar to that encountered in the cell, but this approach does not take into account the growth condition-dependent variation of other factors such as protein copy number, macromolecular interactions and crowding, and turnover rates, all of which are usually unknown or poorly modeled. Here, we have characterized the highly redundant PG DD-CPase system under two sets of conditions by quantifying the pentapeptides in PG as a proxy for total cellular DD-CPase activity. The statistical analysis of the PG composition of 28 single and multiple mutant strains permitted an estimate of the contribution of each DD-CPase in the cell under two different pH conditions (discussed below).

Our approach can potentially unravel the roles of other redundant PG enzymes. For example, analyzing the PG composition of multiple ld-transpeptidase mutants grown under various conditions could determine the contribution of each in the formation of 3-3-crosslinks (47) and the attachment of Braun’s lipoprotein (Lpp) (48). In the case of lytic transglycosylases, which produce 1,6-anhydroMurNAc-containing muropeptides, the PG of mutants lacking multiple enzymes has been analyzed previously, but the PG was isolated from cells grown under standard laboratory conditions (49). It is known that the purified membrane-bound lytic transglycosylase MltA has a pH optimum of 5.2 and higher activity at 30°C than at 37°C (50). Hence, quantifying 1,6-anhydroMurNAc-containing muropeptides in the PG of (multiple) mutant strains grown under different conditions could help to link the activities of individual lytic transglycosylases to particular growth conditions.

### Redundancy of PG DD-CPases in *E. coli*.

We have determined in this study the prominent role of PBP5 at near neutral pH and showed that other DD-CPases can, to some extent, complement the loss of PBP5 at this pH. However, when cells were grown at acidic pH, PBP5 had only negligible activity and, presumably, cellular function. PBP6b, which has a minor role at pH 7.5, provided the main DD-CPase activity at pH 5.0 (see Fig. S1 and S2 in the supplemental material). The key role of PBP6b at low pH was consistent with its *in vitro* enzyme activity (Fig. 3) and stability (Fig. 4). PBP1B made a minor contribution to total DD-CPase activity in cells grown at acidic pH (see Fig. S1C), consistent with its activity *in vitro* (17). The approach utilized here could be applied to other growth variables (e.g., osmolarity, temperature, medium composition) to determine if other DD-CPases are specialized for specific growth conditions. On the basis of our results and of previously published data, we propose that *E. coli* maintains a set of at least eight DD-CPases to ensure that sufficient activity is available across the vast gamut of conditions that support growth of this bacterium. Furthermore, PBP6a appears to be a special case within the family of DD-CPases; consistent with its low activity *in vitro* (38), PBP6a did not contribute significantly to the removal of pentapeptides but rather seemed to protect these from cleavage by other DD-CPases in the cell. Hence, it remains to be elucidated whether PBP6a becomes active only under a particular set of growth conditions or whether it has a cellular function different from those of the other DD-CPases.

### PBP6b is a specialist DD-CPase for growth at acidic pH.

The PBP6b gene, *dacD*, was poorly expressed when cells were grown at pH 7.5 (Fig. 2A), consistent with a protein synthesis rate of well below 50 copies per generation (32). This low cellular concentration and the relative instability of the protein near neutral pH likely explain why the levels of PBP6b in cells grown at pH 7.5 were too low for detection (Fig. 2B). That PBP6b has no major role at pH 7.5 is consistent with the lack of a phenotype upon deletion of *dacD*. However, PBP6b is activated on multiple levels at acidic pH: the protein is expressed at higher levels in the cell (Fig. 2B), has higher specific activity against pentapeptide substrates (Fig. 3C), and has higher thermostability (Fig. 4). The increased enzyme activity is due to the 18-fold-lower *K_m_* at acidic pH than at neutral pH and is consistent with the stronger binding (Table 2) of the substrate analog Bocillin (Fig. 3B). The crystal structure of PBP6b has also been determined (Fig. 4), but comparing the highly conserved active sites of PBP5 and PBP6b does not explain the pH dependency of substrate binding. The only amino acid difference between PBP6b and PBP5 within ~9 Å of the nucleophile is Asn39 in PBP6b, which is an aspartate in PBP5. Based on thermostability data, it would appear that PBP6b becomes destabilized and loses activity at neutral pH, probably due to partial protein unfolding, which does not occur so readily in PBP5. Considering the high redundancy of DD-CPases, most of which are active under other conditions, it could be beneficial for cells growing at pH 7.5 to keep the number of PBP6b molecules low by poor expression and protein unfolding/degradation, ensuring that other DD-CPases are not affected by the poor activity of the enzyme.

### DD-CPase activity is required for proper cell morphology.

Newly made PG is rich in pentapeptide donor peptides, but these are rapidly trimmed by DD-CPases to tetrapeptides (51, 52). Tetrapeptides cannot function as donors for transpeptidation reactions by class A and B PBPs but are donors for ld-transpeptidases which produce 3-to-3-(ld-)cross-links and covalently attach Lpp to PG in *E. coli*. Many DD-CPase mutants have increased pentapeptide content in their PG, and 3-3-crosslinks and attached Lpp levels are both reduced (44, 47, 48); these changes are paralleled by morphological defects, including altered cell diameter and kinked and even branching of cells. Why the absence of DD-CPase activity leads to these defects is not known. Since ld-transpeptidase and Lpp mutants have normal, rod-shaped morphologies, it is likely that the DD-CPase mutant phenotypes are caused by an effect on class A and B PBPs or indirectly by altering the geometry of the septal ring (44). Presumably, the PG synthases or one of their regulators functions improperly when the PG layer has elevated pentapeptide levels, causing malfunctioning or dislocation of the PG synthesis machinery and resulting in the loss of normal cell morphology. As shown in this work, morphological defects can develop in DD-CPase mutants growing at standard or low pH, indicating that the underlying mechanism is not pH dependent. Hence, our data suggest that DD-CPase activity is required for robust growth with normal morphology under different growth conditions, which is the reason why *E. coli* maintains a redundant set of DD-CPases.

## MATERIALS AND METHODS

### Bacterial strains, plasmids, and growth conditions.

Bacteria and plasmids used in this study are listed in Table S2 in the supplemental material Strains with the prefix “SKCS” were constructed for this work; strains with the prefix “CS” were described previously (28, 53). All experiments were carried out at 30°C unless otherwise noted. Overnight cultures were grown in LB and then diluted 1:300 into fresh antibiotic medium (AM3; Difco), also known as Penassay broth. Kanamycin (50 µg/ml) was included when needed.

### Microscopy.

Microscopy was performed with a Zeiss Axio-Cam microscope. Cells (100 µl) were harvested, pelleted, and resuspended in 10 µl of growth medium. A cell suspension (5 µl) was spotted onto an agarose-coated glass slide, the sample was covered with a glass coverslip, and the cells were allowed to settle for 5 to 10 min before imaging.

### SDS-PAGE and Western blotting.

Wild-type *E. coli* and DD-CPase mutant strains were grown overnight in LB before dilution at 1:300 into 50 ml of Penassay broth buffered to either pH 5.0 or pH 7.5. Cell growth was monitored until cultures reached an optical density at 600 nm (OD_600_) of 0.7 to 0.8, at which time the cells were harvested by centrifugation at 10,000 rpm for 10 min at 4°C, in a Sorvall benchtop centrifuge. The cell pellet was resuspended in 10 ml of filter-sterilized 25 mM citric acid-sodium phosphate buffer (pH 5.0 or pH 7.5, as required) containing Halt protease inhibitor (Pierce) (10 µl Halt for every 10 ml of resuspended sample) and DNase (4 µg/ml final concentration). Cells were lysed by using a low-volume, high-shear Microfluidizer LV1 cell disruptor (Microfluidics, Westwood, MA), and cell debris was removed by centrifugation at 10,000 rpm for 10 min at 4°C. The supernatant was collected and denoted the total protein extract. Cell membranes were collected from approximately half of the total protein extract by ultracentrifugation in a Beckman Coulter ultracentrifuge, using a TLA110 rotor, for 1 h at 100,000 rpm at 4°C. The supernatant was stored at 4°C. The membrane pellet was resuspended carefully in 100 µl of the appropriate citric acid-sodium phosphate buffer containing Halt. Proteins in the membrane fraction were quantified with a Micro bicinchoninic acid (BCA) protein assay (Pierce), according to the manufacturer’s protocol. Proteins from the membrane fraction (10 µg) were mixed with 1× Laemmli buffer, boiled for 10 min, and loaded onto a 12% Mini-Protean TGX SDS-PAGE gel (Bio-Rad). The gel was electrophoresed at 100 V for 30 min, at which time the voltage was increased to 120 V until the dye front ran off the bottom of the gel.

For Western blotting, protein bands from the SDS-PAGE gel were transferred to a 0.45-µm-pore-size polyvinylidene difluoride membrane (Millipore) prewetted with methanol. The membrane was rinsed twice (5 min each time) with fresh TBS-T buffer (per liter: 3 g Tris base, 14.4 g glycine, 200 ml methanol, 1 ml Tween 20) and then blocked with 5% nonfat milk–TBS-T for 1 h. The membrane was washed three times with TBS-T for 10 min each time and then labeled with mouse anti-FLAG antibody (Cell Signaling), diluted 1:1,000 in TBS-T–5% bovine serum albumin (BSA), and incubated overnight at 4°C. The membrane was washed in TBS-T three times for 5 min each time and then flooded with a 1:2,000 dilution of anti-mouse horseradish peroxidase (HRP)-linked secondary antibody (Cell Signaling)–5% nonfat milk and incubated for 1 h at 4°C. Following three 5-min washes in TBS-T, the membrane was placed on a stretched sheet of clear plastic wrap, coated with Advansta WesternBright ECL HRP substrate (Bioexpress), incubated for 5 min at room temperature, and then developed. Fresh substrate was prepared for each assay by mixing with 2 ml of each of the two detection reagents.

### Expression and purification of PBP5 and PBP6b.

DNA encoding *E. coli* PBP6b residues 22 to 388 and PBP5 residues 1 to 370 (signal sequences were replaced by a thrombin-cleavable N-terminal oligohistidine tag) was amplified by PCR and cloned into pET28a(+) (Novagen) for overproduction of recombinant proteins. The resulting plasmids (phisDACD and pDACAhis [34]) were verified by sequencing. The PBPs were produced in *E. coli* BL21(DE3) cells grown overnight at 30°C in 1 liter of autoinduction medium (LB medium supplemented with 0.5% glycerol, 0.05% glucose, and 0.2% α-lactose). Cells were harvested by centrifugation for 20 min at 6,200 × *g* and 4°C. The cell pellet was resuspended in 60 ml buffer I (25 mM Tris-HCl, 100 mM NaCl; pH 8.0 for PBP6b or pH 7.5 for PBP5). DNase, protease inhibitor cocktail (Sigma-Aldrich) (1:1,000 dilution), and 100 µM phenylmethylsulfonyl fluoride (Sigma-Aldrich) were added before the cells were lysed by sonication and centrifuged for 1 h at 130,000 × *g* and 4°C. The pellet was resuspended in 20 ml of high-salt buffer II (25 mM Tris-HCl, 10 mM MgCl_2_, 1 M NaCl, 0.02% NaN_3_, 20% glycerol; pH 8.0 for PBP6b, pH 7.5 for PBP5) using a hand homogenizer and incubated with continuous stirring for 2 h at 4°C. The sample was centrifuged for 1 h at 130,000 × *g* and 4°C. ForPBP6b, the extracted supernatant (NaCl extract) was added to 3 ml of nickel-nitrilotriacetic acid (Ni-NTA) Superflow (Qiagen) that had been preequilibrated in buffer II. For PBP5, the pellet was resuspended in 15 ml of extraction buffer (25 mM Tris-HCl, 10 mM MgCl_2_, 1 M NaCl, 0.02% NaN_3_, 2% Triton X-100; pH 7.5) and stirred overnight at 4°C. The sample was clarified by centrifugation for 45 min at 130,000 × *g* and 4°C, and the supernatant (Triton extract) was added to 3 ml of Ni-NTA Superflow (Qiagen) that had been preequilibrated in extraction buffer. For both proteins, the resin was incubated with protein extract for 1.5 h at 4°C with gentle stirring. The resin was poured into a gravity flow column and washed 10 times with 5 ml of buffer II (PBP6b) or extraction buffer (PBP5), both supplemented with 5 mM imidazole, before eluting with buffer II (PBP6b) or extraction buffer (PBP5), both supplemented with 500 mM imidazole.

Eluted proteins were dialyzed into dialysis buffer I (25 mM Tris-HCl, 1 M NaCl, 10 mM EDTA; pH 8.0 for PBP6b or pH 7.5 for PBP5). Thrombin (Novagen) (restriction grade; 10 µl for a 10-ml protein sample) was added, and the mixture was incubated for 16 h at 4°C to cleave the N-terminal His_6_ tag. After 16 h, a further 10-µl aliquot of thrombin was added per 10 ml sample and incubated for a further 8 h. The successful removal of the His_6_ tag was verified by SDS-PAGE and Western blot analysis using monoclonal mouse anti-His-tag antibody (Sigma). Proteins were dialyzed stepwise into dialysis buffer II (10 mM sodium acetate, 10 mM MgCl_2_, 500 mM NaCl, 0.02% NaN_3_; pH 5.0) and diluted 1:5 in buffer C (10°mM sodium acetate, 10 mM MgCl_2_, 0.02% NaN_3_; pH 5.0 [for PBP5, 0.8% Triton X-100]). Proteins were further purified by cation exchange chromatography using a 5 ml HiTrap SP HP column and eluted using a gradient from 100 mM to 1 M NaCl. Eluted proteins were dialyzed into dialysis buffer III (25 mM sodium acetate, 10 mM MgCl_2_, 0.02% NaN_3_, 10% glycerol, 1 M NaCl; pH 5.0). PBP6b was concentrated and further purified by size exclusion chromatography using a HiLoad 16/600 Superdex 200-pg column and dialysis buffer II. Afterward, PBP6b was dialyzed into dialysis buffer III (25 mM sodium acetate, 10 mm MgCl_2_, 1 M NaCl, 10% glycerol, 0.02% NaN_3_), flash-frozen in liquid nitrogen, and stored until further use at −80°C.

### Purification of PBP6bΔC.

For structural studies, the DNA coding for residues 1 to 353 of PBP6B, which thus lacked both the signal peptide and the C-terminal amphipathic helix, was cloned by PCR into a modified pET15b derivative that encoded a tobacco etch virus (TEV)-cleavable His_6_ tag at the N terminus of the encoded protein, PBP6bΔC. The overexpression plasmid was transformed into *E. coli* BL21(DE3) and grown at 37°C. Once the culture had reached an OD_600_ of 0.5 to 0.7, PBP6bΔC expression was induced with 1 mM IPTG (isopropyl-β-d-thiogalactopyranoside) and the culture was grown for a further 21 h at 22°C. The cell culture was harvested by centrifugation, and the pellet was resuspended in a buffer consisting of 50 mM Tris-HCl (pH 7.5), 500 mM NaCl, 20 mM imidazole supplemented with one EDTA-free protease inhibitor tablet (Roche), and DNase I to reach a final concentration of 4 µg/ml. The cells were lysed using a One Shot cell disruptor (Constant Systems) at 172 MPa, and the cell debris was removed by centrifugation (40,000 × *g* at 4°C for 30 min). The supernatant was applied to an Ni-charged HisTrap FF column (GE Healthcare) preequilibrated in 50 mM Tris-HCl (pH 7.5), 500 mM NaCl, 20 mM imidazole. After loading the sample, the column was washed with a buffer consisting of 50 mM Tris-HCl (pH 7.5), 500 mM NaCl, and 20 mM imidazole before the bound proteins were gradient eluted against increasing imidazole concentrations from a buffer consisting of 50 mM Tris-HCl (pH 7.5), 500 mM NaCl, and 800 mM imidazole. The His_6_ tag was removed by overnight TEV cleavage (1 µg TEV per mg PBP6bΔC) prior to size exclusion chromatography performed using a Superdex 75 HiLoad 16/60 column (GE Healthcare) equilibrated in a buffer consisting of 25 mM Tris-HCl (pH 7.5) and 150 mM NaCl. The purified protein was flash-frozen in liquid nitrogen and stored at −80°C.

### Crystallization and X-ray crystallography of PBP6bΔC.

For crystallization, purified PBP6bΔC was lysine methylated overnight according to the instructions of the manufacturer (Jena Biosciences). This sample was subsequently loaded onto a Superdex 75 HiLoad 16/60 column (GE Healthcare) equilibrated in a buffer consisting of 25 mM Tris-HCl (pH 7.5), 150 mM NaCl, and 5 mM dithiothreitol (DTT). The proteins that eluted in the void fraction were separated from those that eluted as a monomer and discarded, and the remaining fraction was concentrated to 13 mg/ml. The pseudosubstrate diacetyl-l-Lys-d-Ala-d-Ala was added to reach a 2 mM final concentration before 100-nl sitting-drop vapor diffusion commercial crystallization screens were set up with a Mosquito (TTP Labtech) pipetting robot. Crystals took 3 months to grow in a crystallization solution consisting of 20% polyethylene glycol (PEG) 3000 and 0.1 M sodium citrate (pH 5.5), and the PBP6bΔC crystals were cryoprotected by adding 25% PEG 400 to the mother liquor before flash-freezing in liquid nitrogen was performed. Diffraction data were collected on beamline I02 of the Diamond synchrotron light source, indexed in XDS (54), and scaled and reduced in AIMLESS (55). The crystals belong to space group P1, with two molecules of PBP6bΔC in the unit cell. The structure was solved by molecular replacement using PHASER (56) and the structure of *E. coli* PBP5 (PDBid 1NZO [37]) as the search model. The structure of PBP6bΔC was refined in REFMAC5 (57) interspersed with rounds of manual rebuilding in COOT (58) until the refinement reached convergence. Data collection and refinement statistics are summarized in Table S3 in the supplemental material.

### Differential scanning fluorimetry.

Using the fluorescent dye SYPRO Orange (Sigma Aldrich), a thermal shift assay (59) was performed to assess the thermal stability of PBP6bΔC at pH 5.0 and pH 7.5. Solutions of PBP6b and PBP6bΔC at a 1 µM concentration in the presence of 5× SYPRO Orange were prepared at the desired pH (using citric acid or sodium phosphate buffer), and 40 µl of each solution was added to thin-wall PCR tubes. The tubes were heated in a Rotor Gene 6000 quantitative PCR (qPCR) machine (Corbett Life Sciences) from 25°C to 99°C at a rate of 1°C per min. Fluorescence changes were monitored with excitation and emission wavelengths of 492 and 610 nm, respectively. *T_m_* values were compared for each sample, with a higher *T_m_* indicative of higher thermal stability. The experiments were carried out in triplicate, and nonlinear regression analysis was performed with Rotor Gene 6000 proprietary software.

### Bocillin-binding assay.

The activity of PBP6b was tested in an assay using the fluorescent penicillin V derivate Bocillin-FL (Life Technologies) (60). Ten micrograms of PBP6b was incubated with 1 ng/µl of bocillin at 37°C. Reactions were performed in the presence of 10 mM MgCl_2_ at pH 5.0 (10 mM sodium acetate) and pH 7.5 (10 mM HEPES). Samples were taken after 0, 0.5, 1, 2, 5, 10, 15, 30, and 40 min; the reactions were stopped by addition of 4× SDS-PAGE sample buffer and by boiling for 30 min at 100°C. The proteins were separated by 12% SDS-PAGE before the bocillin-PBP complexes were detected with a GE Healthcare Typhoon laser scanner (excitation laser, 488 nm; emission filter, 520 nm BP 40; photomultiplier tube [PMT] voltage, 600 V), and proteins were subsequently stained with Coomassie blue.

### Activity of PBP6b and PBP5 against PG.

PBP6b and PBP5 were incubated at different concentrations (0.01, 0.03. 0.1, 0.3, or 1 µM) with pentapeptide-rich PG from *E. coli* CS703/1 or at a concentration of 0.3 µM with glycine and pentapeptide-rich sacculi from *C. crescentus* CB15N. The reactions were performed for 1 h in a Thermomixer at 37°C and 700 rpm in a total volume of 50 µl and buffered either at pH 7.5 (25 mM HEPES, 10 mM MgCl_2_, 150 mM NaCl) or at pH 5.0 (25 mM sodium acetate, 10 mM MgCl_2_, 150 mM NaCl). The enzymes were inactivated by boiling for 10 min at 100°C. The samples were digested with cellosyl at 37°C and 700 rpm overnight, followed by heat inactivation for 10 min at 100°C. The samples were centrifuged for 15 min at 17.000 × *g*. The muropeptides present in the supernatant were reduced with sodium borohydride and separated by HPLC according to published procedures (61). Muropeptide profiles were quantified with Laura software (Lab Logic Systems Ltd.).

### Spectrophotometric d-alanine release assay for *E. coli* PBP6b dd-carboxypeptidase activity.

The DD-CPase activity of PBP6b under different pH conditions was determined by a modified spectrophotometric assay (62) which measures the release of d-alanine, using a Biochrom Libra S22 UV-visible light (UV/Vis) spectrophotometer and matched quartz cuvettes (Starna Scientific Ltd.) (path length, 10 mm). The substrate diacetyl-l-Lys-d-Ala-d-Ala was kindly provided by Mohammed Terrak, University of Liege. In this assay, the d-amino acid oxidase (DAAO; Sigma Aldrich) deaminates the released d-alanine, generating pyruvate and hydrogen peroxide. The latter is reduced to H_2_O by horseradish peroxidase (HRP, Sigma Aldrich) using Amplex Red (Invitrogen) as an electron donor, producing resorufin, which can be quantified spectrophotometrically at 563 nm. Because the published continuous assay did not work at pH 5.0, we modified the assay protocol for endpoint measurements. For this, 1 µM PBP6b was incubated at 37°C in a final volume of 500 µl using 50 mM sodium acetate (pH 5.0) or 50 mM HEPES (pH 7.5) with various concentrations of diacetyl-l-Lys-d-Ala-d-Ala. After different time periods, 50-µl samples were withdrawn from the sample and the reactions were stopped by boiling for 20 min at 100°C. After a short centrifugation step for 5 min at 17.000 × *g* was performed, the samples were stored at −20°C until further use. The released d-alanine was quantified by adding the withdrawn sample to 200 µl of a mixture consisting of 50 mM HEPES-NaOH, 10 mM MgCl_2_ (pH 7.5), 50 µM Amplex Red, 75.5 µg/ml DAAO, and 54.5 µg/ml HRP. Samples were incubated for 60 min at 37°C, and the absorbance at 563 nm was measured. Standard samples contained d-alanine at known concentrations.

The kinetic constants *K_m_* and *V*_max_ corresponding to the CPase activity of PBP6b were calculated using GraphPad Prism 6 software. The initial velocities of PBP6b CPase activity versus substrate concentrations were fitted by nonlinear regression to equation 1:

$$V(0)=\frac{Vmax\;[S]}{Km+[S]}$$

### Peptidoglycan isolation and analysis.

Penassy broth (Difco antibiotic medium 3) adjusted to pH 5.0 or 7.5 with maleic acid or NaOH was used for growing cells to an OD_578_ of 0.6 at 30°C. The pH value did not change during growth. PG was prepared and analyzed according to a method published previously (61).

### Flow cytometry.

Wild-type *E. coli* and the DD-CPase mutants were grown overnight in LB at 30°C. The cells were diluted 1:300 into 5 ml of Penassay broth buffered at pH 5.0 or pH 7.5. Cells were grown until the OD_600_ reached 0.7 to 0.8, and cells from 1 ml of culture were pelleted by centrifugation, washed twice, and then diluted 1:20 into filter-sterilized 25 mM citric acid-sodium phosphate buffer at the appropriate pH. Cells were analyzed by using a BD LSRFortessa flow cytometer (BD Biosciences), housed in the University of Arkansas for Medical Sciences (UAMS) flow cytometry core.

### Statistical analyses.

Multivariable linear regression models were fitted to estimate the association between the presence or absence of each PBP (coded as a 0/1 predictor variable) and the resulting muropeptide level (i.e., the response variable). The coefficient estimates from these models represent the influence of each PBP mutant on the amounts of each muropeptide.

### Protein structure accession number.

Atomic coordinates and structure factors have been deposited at the PDB with accession code 5FSR.

## SUPPLEMENTAL MATERIAL

Figure S1  PBP-associated changes (±95% confidence intervals) in penta- and tetrapeptide-containing muropeptides in all *E. coli* strains tested (*n* = 28), including those with and without PBP5. Bacteria were grown in media buffered to pH 7.5 (A and B) or pH 5.0 (C and D), and muropeptide compositions were determined by HPLC. The plotted values are the changes in muropeptide levels associated with the presence of each PBP compared to absence of that PBP, and were computed using multivariable linear regression as described in Materials and Methods. DD-CPase activity decreases pentapeptides and increases tetrapeptides. Download Figure S1, PDF file, 0.3 MB

Figure S2  PBP-associated changes (±95% confidence intervals) in penta- and tetrapeptide-containing muropeptides in *E. coli* strains lacking PBP5 (*n* = 26). Bacteria were grown in media buffered to pH 7.5 (A and B) or pH 5.0 (C and D), and muropeptide compositions were determined by HPLC. The plotted values are the changes in muropeptide levels associated with the presence of each PBP compared to absence of that PBP, and were computed using multivariable linear regression as described in Materials and Methods. DD-CPase activity decreases pentapeptides and increases tetrapeptides. Download Figure S2, PDF file, 0.3 MB

Figure S3  Activity of PBP6b against pentapeptide-rich PG at pH 5.0 and 7.5. PBP6b was incubated with pentapeptide-rich PG from *E. coli* CS703-1 at pH 5.0 or 7.5 at the concentration indicated, and the muropeptide composition was analyzed as described in Materials and Methods. Bar, 500 mAU. Muropeptides are numbered as in Fig. 1, which also shows the structures. 1, Tri; 2, Tetra; 3, Penta; 4, TetraTetra; 5, TetraPenta. Download Figure S3, PDF file, 0.4 MB

Figure S4  Activity of PBP5 against pentapeptide-rich PG at pH 5.0 and 7.5. PBP5 was incubated with pentapeptide-rich PG from *E. coli* CS703-1 at pH 5.0 or 7.5 at the concentration indicated, and the muropeptide composition was analyzed as described in Materials and Methods. Bar, 500 mAU. Muropeptides are numbered as in Fig. 1, which also shows the structures. 1, Tri; 2, Tetra; 3, Penta; 4, TetraTetra; 5, TetraPenta. Download Figure S4, PDF file, 0.4 MB

Figure S5  Activity of PBP6b and PBP5 against PG from *Caulobacter crescentus* at pH 5.0 and 7.5. (A) Pentapeptide-rich PG from *C. crescentus* was incubated with PBP6b (0.3 µM) or PBP5 (0.3 µM) at pH 5.0 or pH 7.5, and the muropeptide composition was analyzed by HPLC as described in Materials and Methods. Bar, 500 mAU. Muropeptides are numbered as in Fig. 1. Peaks A and B are the glycine containing muropeptides PentaGly5 and TetraPentaGly5, respectively. (B) Quantification of the major muropeptides from the HPLC profiles. The values are mean ± variation of 2 independent experiments. Download Figure S5, PDF file, 0.3 MB

Figure S6  Sequence alignment of full-length PBP6b with PBP5 (UniProt codes: P33013 and P0AEB2, respectively). Domains are shown as colored bars: CPase domain in green and the C-terminal domain in dark blue. Active site sequences motifs essential for catalysis are highlighted in green. Secondary structure based on the solved crystal structure of PBP6b is depicted above the corresponding residues as blue arrows (beta-sheets) and pink cylinders (alpha-helices). The C-terminal amphiphilic helix is shown in orange. Sequence alignment was generated and annotated using Clustal Omega (Sievers et al. [2011] Mol Systems Biol **7**: 539) and Aline (Bond and Schüttelkopf [2009] acta Cryst **D65**: 510 to 512), respectively. Download Figure S6, PDF file, 0.5 MB

Table S1  PG composition of PBP mutant strains (separate Excel file). Table S1, XLSX file, 3.8 MB

Table S2  Bacterial strains and plasmids. Table S2, PDF file, 0.3 MB

Table S3  Crystallographic parameters. Table S3, PDF file, 0.3 MB
