# Supplementary material for: The Redundancy of Peptidoglycan Carboxypeptidases Ensures Robust Cell Shape Maintenance in Escherichia coli
Source: mBio. 2016 Jun 21;7(3):e00819-16. doi: 10.1128/mBio.00819-16 (PMC4916385; doi:10.1128/mBio.00819-16)
Supplement: Figure S2 — PBP-associated changes (±95% confidence intervals) in penta- and tetrapeptide-containing muropeptides in E. coli strains lacking PBP5 (n = 26). Bacteria were grown in media buffered to pH 7.5 (A and B) or pH 5.0 (C and D), and muropeptide compositions were determined by HPLC. The plotted values are the changes in muropeptide levels associated with the presence of each PBP compared to absence of that PBP, and were computed using multivariable linear regression as described in Materials and Methods. DD-CPase activity decreases pentapeptides and increases tetrapeptides. Download [file mbo003162862sf2.pdf]

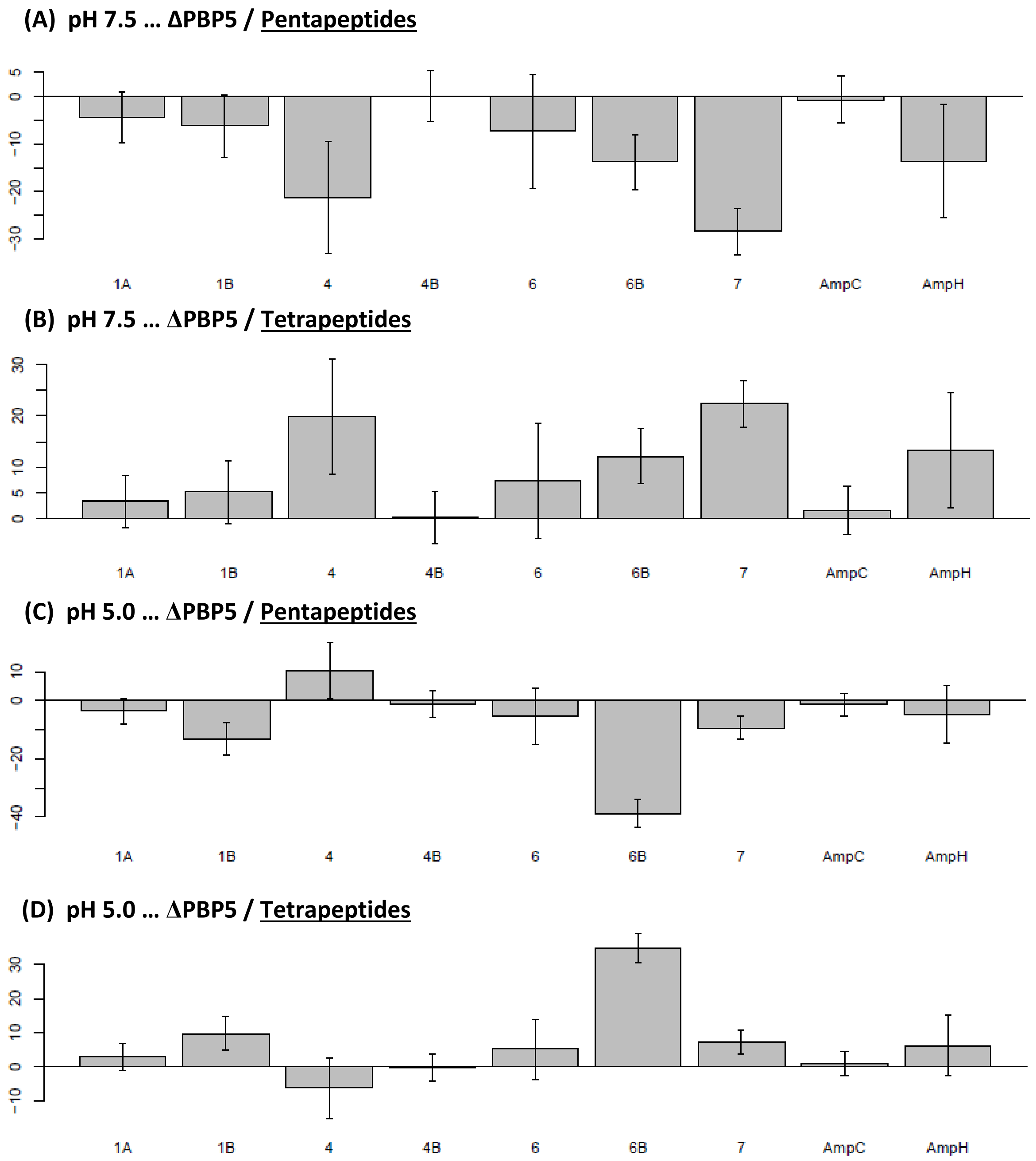

Figure S2. PBP-associated changes (± 95% confidence intervals) in penta- and tetrapeptide-containing muropeptides in *E. coli* strains lacking PBP5 (n = 26). Bacteria were grown in media buffered to pH 7.5 (A and B) or pH 5.0 (C and D), and muropeptide compositions were determined by HPLC. The plotted values are the changes in muropeptide levels associated with the presence of each PBP compared to absence of that PBP, and were computed using multivariable linear regression as described in Materials and Methods. DD-CPase activity decreases pentapeptides and increases tetrapeptides.
