# Supplementary material for: The Redundancy of Peptidoglycan Carboxypeptidases Ensures Robust Cell Shape Maintenance in Escherichia coli
Source: mBio. 2016 Jun 21;7(3):e00819-16. doi: 10.1128/mBio.00819-16 (PMC4916385; doi:10.1128/mBio.00819-16)
Supplement: Figure S3 — Activity of PBP6b against pentapeptide-rich PG at pH 5.0 and 7.5. PBP6b was incubated with pentapeptide-rich PG from E. coli CS703-1 at pH 5.0 or 7.5 at the concentration indicated, and the muropeptide composition was analyzed as described in Materials and Methods. Bar, 500 mAU. Muropeptides are numbered as in Fig. 1, which also shows the structures. 1, Tri; 2, Tetra; 3, Penta; 4, TetraTetra; 5, TetraPenta. Download [file mbo003162862sf3.pdf]

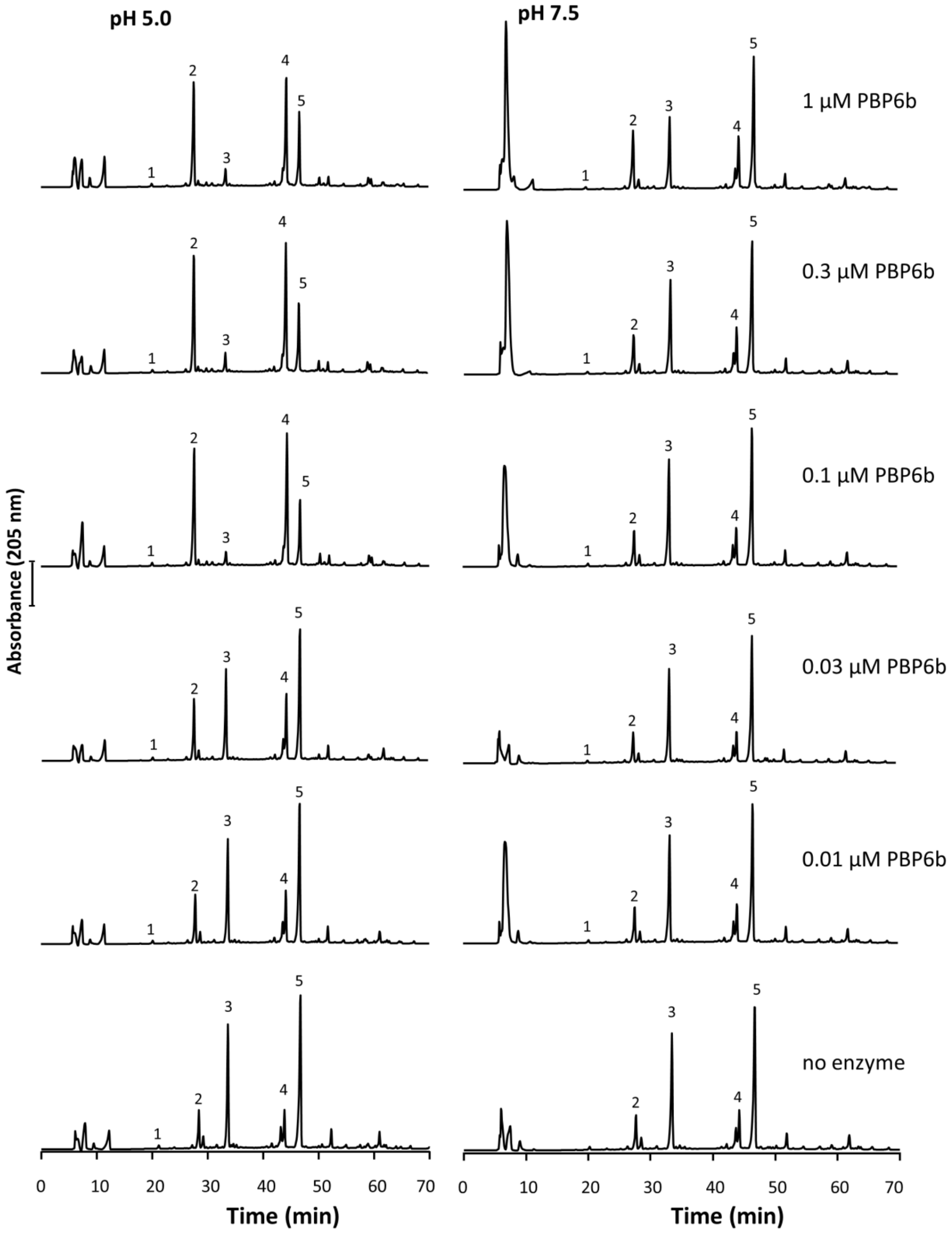

Figure S3. Activity of PBP6b against pentapeptide-rich PG at pH 5.0 and 7.5. PBP6b was incubated with pentapeptide-rich PG from *E. coli* CS703-1 at pH 5.0 or 7.5 at the concentration indicated, and the muropeptide composition was analysed as described in Materials and Methods. Bar, 500 mAU. Muropeptides are numbered as in Fig. 1, which also shows the structures. 1, Tri; 2, Tetra; 3, Penta; 4, TetraTetra; 5, TetraPenta.
