# Supplementary material for: The Redundancy of Peptidoglycan Carboxypeptidases Ensures Robust Cell Shape Maintenance in Escherichia coli
Source: mBio. 2016 Jun 21;7(3):e00819-16. doi: 10.1128/mBio.00819-16 (PMC4916385; doi:10.1128/mBio.00819-16)
Supplement: Figure S5 — Activity of PBP6b and PBP5 against PG from Caulobacter crescentus at pH 5.0 and 7.5. (A) Pentapeptide-rich PG from C. crescentus was incubated with PBP6b (0.3 µM) or PBP5 (0.3 µM) at pH 5.0 or pH 7.5, and the muropeptide composition was analyzed by HPLC as described in Materials and Methods. Bar, 500 mAU. Muropeptides are numbered as in Fig. 1. Peaks A and B are the glycine containing muropeptides PentaGly5 and TetraPentaGly5, respectively. (B) Quantification of the major muropeptides from the HPLC profiles. The values are mean ± variation of 2 independent experiments. Download [file mbo003162862sf5.pdf]

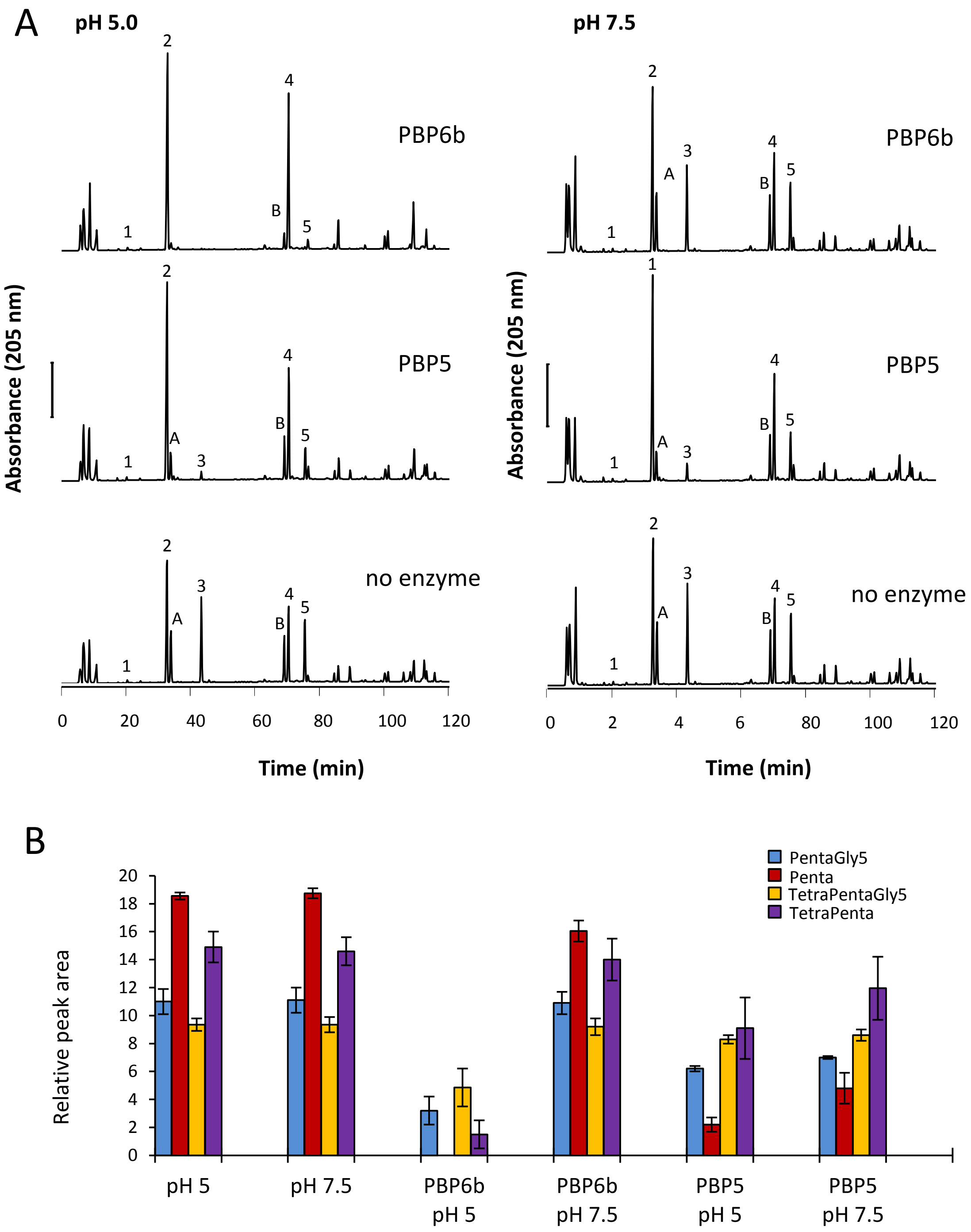

Figure S5. Activity of PBP6b and PBP5 against PG from *Caulobacter crescentus* at pH 5.0 and 7.5. (**A**) Pentapeptide-rich PG from *C. crescentus* was incubated with PBP6b (0.3 µM) or PBP5 (0.3 µM) at pH 5.0 or pH 7.5, and the muropeptide composition was analyzed by HPLC as described in Materials and Methods. Bar, 500 mAU. Muropeptides are numbered as in Fig. 1. Peaks A and B are the glycine containing muropeptides PentaGly5 and TetraPentaGly5, respectively. (**B**) Quantification of the major muropeptides from the HPLC profiles. The values are mean ± variation of 2 independent experiments.
