# Supplementary material for: The Redundancy of Peptidoglycan Carboxypeptidases Ensures Robust Cell Shape Maintenance in Escherichia coli
Source: mBio. 2016 Jun 21;7(3):e00819-16. doi: 10.1128/mBio.00819-16 (PMC4916385; doi:10.1128/mBio.00819-16)
Supplement: Figure S6 — Sequence alignment of full-length PBP6b with PBP5 (UniProt codes: P33013 and P0AEB2, respectively). Domains are shown as colored bars: CPase domain in green and the C-terminal domain in dark blue. Active site sequences motifs essential for catalysis are highlighted in green. Secondary structure based on the solved crystal structure of PBP6b is depicted above the corresponding residues as blue arrows (beta-sheets) and pink cylinders (alpha-helices). The C-terminal amphiphilic helix is shown in orange. Sequence alignment was generated and annotated using Clustal Omega (Sievers et al. [2011] Mol Systems Biol 7: 539) and Aline (Bond and Schüttelkopf [2009] acta Cryst D65: 510 to 512), respectively. Download [file mbo003162862sf6.pdf]

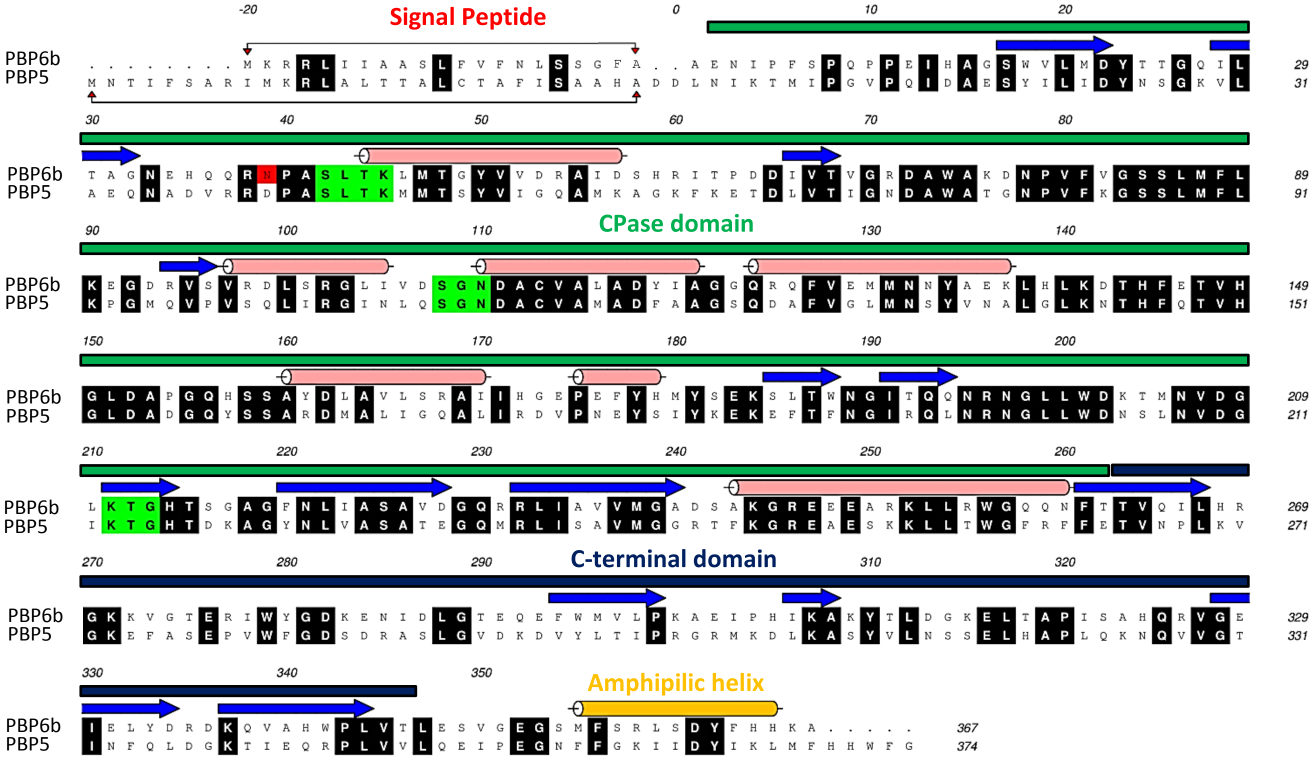

Figure S6. Sequence alignment of full-length PBP6b with PBP5 (UniProt codes: P33013 and P0AEB2, respectively). Domains are shown as coloured bars: CPase domain in green and the C-terminal domain in dark blue. Active site sequences motifs essential for catalysis are highlighted in green. Secondary structure based on the solved crystal structure of PBP6b is depicted above the corresponding residues as blue arrows (beta-sheets) and pink cylinders (alpha-helices). The C-terminal amphiphilic helix is shown in orange. Sequence alignment was generated and annotated using Clustal Omega [Sievers *et al*. (2011) Mol Sys Biol **7**: 539] and Aline [Bond and Schüttelkopf (2009) Acta Cryst **D65**: 510-512], respectively.
