# Supplementary material for: The Redundancy of Peptidoglycan Carboxypeptidases Ensures Robust Cell Shape Maintenance in Escherichia coli
Source: mBio. 2016 Jun 21;7(3):e00819-16. doi: 10.1128/mBio.00819-16 (PMC4916385; doi:10.1128/mBio.00819-16)
Supplement: Table S2 — Bacterial strains and plasmids. [file mbo003162862st2.pdf]

Table S2. Bacterial strains and plasmids.

Strain / Plasmid	Genotype / Reference
**Strains**	
CS109	W1485 *rpoS(katF) rph*
CS204-1	CS109 *dacA::res pbpG::res*
CS219-1	CS109 *dacA::res dacB::res*
CS315-1	CS109 *dacA::res pbpG::res dacB::res*
CS442-3	CS109 *dacC::res ampH::res dacA::res dacB::res*
CS617-1K	CS109 *mrcA::res ampC::res dacC::res pbpG::res ampH::res dacB::res-kan-res*
CS618-1K	CS109 *mrcA::res ampC::res dacC::res pbpG::res ampH::res dacA::res-kan-res*
CS619-1K	CS109 *mrcA::res ampC::res dacA::res dacB::res dacC::res ampH::res-kan-res*
CS806-1K	CS109 *ampC::res dacC::res pbpG::res ampH::res dacA::res dacB::res mrcA::res dacD::res-kan-res*
SKCS66-1	CS109 *dacC::res ampH::res dacA::res dacB::res mrcB::res dacD::res pbp4b::frt-kan-frt*
SKCS67-1	CS109 *dacC::res ampH::res dacA::res dacB::res mrcA::res dacD::res pbp4b::frt-kan-frt*
SKCS68-1	CS109 *dacC::res ampH::res dacA::res dacB::res pbp4b::frt-kan-frt*
SKCS69-1	CS109 *dacC::res ampH::res dacA::res dacB::res dacD::res-kan-res*
SKCS72-1	CS109 *mrcA::res ampC::res dacC::res pbpG::res ampH::res dacA::res dacB::res pbp4b::frt-kan-frt*
SKCS77-1	CS109 *ampC::res dacC::res pbpG::res ampH::res dacA::res dacB::res mrcA::res dacD::res pbp4b::res-kan-res*
SKCS78-1	CS109 *ampC::res dacC::res pbpG::res ampH::res dacA::res dacB::res pbp4b::frt-kan-frt*
SKCS80-1	CS109 *dacC::res ampH::res dacA::res dacB::res mrcA::res dacD::res pbp4b*::*frt pbpG::res-kan-res*
SKCS81-1	CS109 *dacC::res ampH::res dacA::res dacB::res mrcB::res dacD::res pbp4b*::*frt pbpG::res-kan-res*
SKCS82-1	CS109 *dacC::res ampH::res dacA::res dacB::res mrcB::res dacD::res pbp4b*::*frt ampC::res-kan-res*
SKCS87-1	CS109 *dacC::res ampH::res dacA::res dacB::res mrcB::res dacD::res pbp4b*::*frt ampC::res pbpG::res-kan-res*
SKCS88-1	CS109 *dacC::res ampH::res dacA::res dacB::res dacD::res pbp4b*::*frt pbpG::res-kan-res*
SKCS89-1	CS109 *dacC::res ampH::res dacA::res dacB::res dacD::res pbp4b*::*frt ampC::res-kan-res*
SKCS97-1	CS109 *dacC::res ampH::res dacA::res dacB::res mrcA::res dacD::res pbp4b*::*frt ampC::res-kan-res*
SKCS99-1	CS109 *ampC::res dacC::res pbpG::res ampH::res dacA::res dacB::res mrcB::res dacD::res-kan-res*
SKCS100-1	CS109 *dacC::res ampH::res dacA::res dacB::res dacD::res pbp4b*::*frt ampC::res pbpG::res-kan-res*
SKCS127-1	CS109 *dacA::res pbpG::res dacB::res dacD::res*
SKCS132-1	CS109 *dacA::res dacB::res dacD::res-kan-res*
SKCS133-1	CS109 *dacA::res pbpG::res dacD::res-kan-res*
SKCS138-1	CS109 *dacA*-FLAG::*frt-kan-frt*
SKCS139-1	CS109 *dacD*-FLAG::*frt-kan-frt*
SKCS140-1	CS109 *dacA::res pbpG::res dacB::res dacD*-FLAG::*frt-kan-frt*
SKCS143-1	CS109 *dacD::sfgfp*
SKCS144-1	CS109 *dacA::res pbpG::res dacB::res dacD::sfgfp*
**Plasmids**[a]	
pLP18-kan	P_{BAD} vector (1)
pLP516	P_{BAD}-*dacA*, kan (1)
pLP517	P_{BAD}-*dacA*$^{(S44G)}$, kan (1)
pLP653	P_{BAD}-*dacD*, kan (1)
pLP655	P_{BAD}-*dacD*$^{(S63A)}$, kan (1)
pLPKC604	P_{BAD}-*dacC*, kan (1)
pLP606	P_{BAD}-*dacC*$^{(S66A)}$, kan (1)
pDACAhis	pET28a(+) derivative, for overexpression of His-PBP5 (2)
phisDACD	pET28a(+) derivative, that encodes PBP6b (1-366), this work
pET15bTEV	pET15b derivative, that encodes PBP6bΔC (1-353) for crystallographic studies, this work

[a] References: (1) Potluri *et al*. (2012) Mol Microbiol **84:**203-224. (2) Potluri *et al*. (2010) Mol Microbiol **77**:300-323.
