# Supplementary material for: The Redundancy of Peptidoglycan Carboxypeptidases Ensures Robust Cell Shape Maintenance in Escherichia coli
Source: mBio. 2016 Jun 21;7(3):e00819-16. doi: 10.1128/mBio.00819-16 (PMC4916385; doi:10.1128/mBio.00819-16)
Supplement: Table S3 — Crystallographic parameters. [file mbo003162862st3.pdf]

Table S3. Crystallographic parameters.

Space group	*P*1
Unit-cell parameters	
a, b, c (Å)	35.25, 56.00, 108.14
α, β, γ (°)	104.88, 95.24, 90.60
Resolution[a] (Å)	43.59 – 2.40 (2.49 – 2.40)
No. reflections recorded	56169 (5123)
Unique reflections	29631 (2986)
Completeness (%)	95.2 (91.2)
$\langle I/\sigma I \rangle$	5.1 (2.3)
Wilson B ($Å^2$)	25.0
Redundancy	1.9
Radiation source	Diamond Light Source (I02)
Wavelength (Å)	0.979030
No. of residues	Chain A: 318, Chain B: 317
No. of waters	171
R_{merge}[b] (%)	9.2 (20.2)
R_{work}[c] (%)	25.9
R_{free}[d] (%)	30.2
Average B-factor ($Å^2$)	
Protein	Chain A: 35.7, Chain B: 36.5
Waters	30.4
Cruickshank DPI[e] (Å)	0.32
Ramachandran plot	
Most favoured (%)	93.5
Additional allowed (%)	6.2
Outliers (%)	0.3
R.m.s.d on ideal values[f]	
Bond lengths (Å)	0.01
Bond angle (°)	1.60

a Values in parentheses refer to the highest resolution shell.

b $R_{merge} = \sum_{hkl}\sum_i |I_i(hkl) - \langle I(hkl)\rangle| / \sum_{hkl}\sum_i I_i(hkl)$; where $I_i(hkl)$ is the intensity of the *i*th measurement of reflection *hkl* and $\langle I(hkl)\rangle$ is the mean value of $I_i(hkl)$ for all *I* measurements.

c $R_{work} = \sum_{hkl}||F_o|-|F_c||/\sum|F_o|$, where F_o is the observed structure factor and F_c is the calculated structure factor.

d R_{free} is the same as R_{cryst} except calculated with a subset, 5%, of data that are excluded from refinement calculations.

e Diffraction precision index, Cruickshank *et al*. (1999) Acta Crystallogr D Biol Crystallogr **55**:583-601.

f Engh and Huber (1991) Acta Crystallogr A Found Crystallogr **47**:392-400.
